# Advances in Understanding Carboxysome Assembly in *Prochlorococcus* and *Synechococcus* Implicate CsoS2 as a Critical Component

**DOI:** 10.3390/life5021141

**Published:** 2015-03-27

**Authors:** Fei Cai, Zhicheng Dou, Susan L. Bernstein, Ryan Leverenz, Eric B. Williams, Sabine Heinhorst, Jessup Shively, Gordon C. Cannon, Cheryl A. Kerfeld

**Affiliations:** 1 Department of Plant and Microbial Biology, University of California, Berkeley, CA 94720, USA; E-Mails: fcai@lbl.gov (F.C.); slbernstein@lbl.gov (S.L.B.); 2 Physical Biosciences Division, Lawrence Berkeley National Laboratory, Berkeley, CA 94720, USA; 3 Department of Chemistry and Biochemistry, The University of Southern Mississippi, Hattiesburg, MS 39406-5043, USA; E-Mails: zdou@umich.edu (Z.D.); Eric.Williams@usm.edu (E.B.W.); sabine.heinhorst@usm.edu (S.H.); Gordon.Cannon@usm.edu (G.C.C.); 4 MSU-DOE Plant Research Laboratory, Michigan State University, East Lansing, MI 48824, USA; E-Mail: rlleverenz@lbl.gov; 5 Department of Genetics and Biochemistry, Clemson University, Clemson, SC 29634, USA; E-Mail: shy53911@bellsouth.net

**Keywords:** CO_2_ concentrating mechanism (CCM), carboxysome, RuBisCO, CsoS2, assembly, intrinsically disordered protein, peptide array

## Abstract

The marine *Synechococcus* and *Prochlorococcus* are the numerically dominant cyanobacteria in the ocean and important in global carbon fixation. They have evolved a CO_2_-concentrating-mechanism, of which the central component is the carboxysome, a self-assembling proteinaceous organelle. Two types of carboxysome, α and β, encapsulating form IA and form IB d-ribulose-1,5-bisphosphate carboxylase/oxygenase, respectively, differ in gene organization and associated proteins. In contrast to the β-carboxysome, the assembly process of the α-carboxysome is enigmatic. Moreover, an absolutely conserved α-carboxysome protein, CsoS2, is of unknown function and has proven recalcitrant to crystallization. Here, we present studies on the CsoS2 protein in three model organisms and show that CsoS2 is vital for α-carboxysome biogenesis. The primary structure of CsoS2 appears tripartite, composed of an N-terminal, middle (M)-, and C-terminal region. Repetitive motifs can be identified in the N- and M-regions. Multiple lines of evidence suggest CsoS2 is highly flexible, possibly an intrinsically disordered protein. Based on our results from bioinformatic, biophysical, genetic and biochemical approaches, including peptide array scanning for protein-protein interactions, we propose a model for CsoS2 function and its spatial location in the α-carboxysome. Analogies between the pathway for β-carboxysome biogenesis and our model for α-carboxysome assembly are discussed.

## 1. Introduction

Cyanobacteria play an important role in global carbon fixation. In particular, the numerically dominant open ocean cyanobacteria *Synechococcus* and *Prochlorococcus* contribute a significant fraction of total primary production [1,2,3]. Cyanobacteria have evolved a CO_2_ concentrating mechanism (CCM) to enhance the CO_2_ fixation activity of the enzyme ribulose 1,5-bisphosphate carboxylase/oxygenase (RuBisCO), thereby improving photosynthetic performance. The central component of the CCM is a self-assembling proteinaceous organelle, the carboxysome. There are two types of carboxysome, α and β, encapsulating form IA and form IB RuBisCO, respectively. The two carboxysome types also differ in associated carbonic anhydrase (CA) and core proteins. Indeed, the only known protein homologs shared by the α- and β-carboxysome are RuBisCO and the shell proteins.

**Figure 1:**
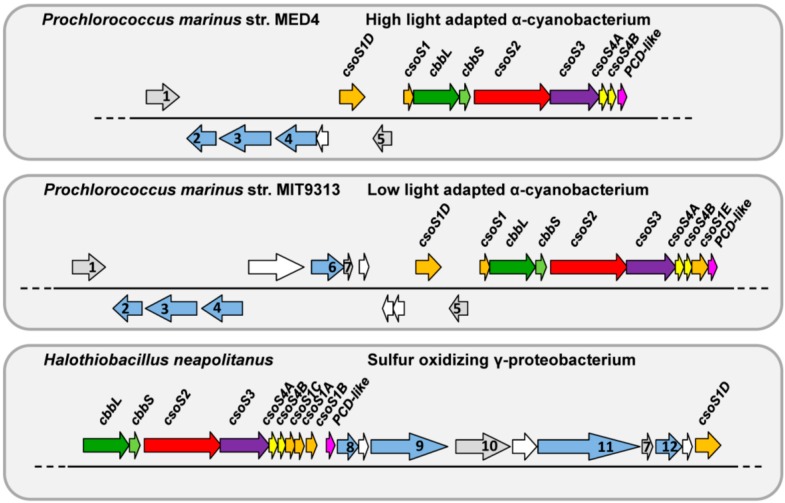
Schematic of α-carboxysome gene organization in three model organisms. Locus boundaries are based on the LoClass algorithm for BMCclassification [4]. Conserved *cso* genes are color-coded: Bacterial Microcompartment domain (BMC; pfam00936)-containing genes (*csoS1s*) in orange; RuBisCO large and small subunits (*cbbL/S*) in dark and light green, respectively; *csoS2* in red; carbonic anhydrase (*csoS3*) in purple; genes belong to pfam03319 (*csoS4A/B*) in yellow; and genes encoding pterin-4 alpha-carbinolamine dehydratase-like protein (PCD-like) in magenta. Gray-blue genes are shared within the BMC locus subtype; gray genes are shared with at least one other BMC locus type; white genes indicate that this gene is not considered part of the locus [4]. Annotations for gray-blue or gray coded genes are as following: **1**. *por* (protochlorophyllide oxidoreductase); **2**. *chlL* (light-independent protochlorophyllide reductase iron-sulfur ATP-binding protein); **3**. *chlB* (light-independent protochlorophyllide reductase subunit B); **4**. *chlN* (light-independent protochlorophyllide reductase subunit N); **5**. HAM1; **6**. *sbtA* (high-affinity bicarbonate transporter); **7**. *sbtB* (or annotated as nitrogen regulatory protein P-II); **8**. *cbiA* (cobyrinic acid a,c-diamide synthase); **9**. *vwfA* (von Willebrand factor type A); **10**. *nuoL* (NADH-quinone oxidoreductase subunit L); **11**. conserved gene with unknown function DUF2309 and **12**. *cbbQ* (a putative catalytic chaperone of RuBisCO). Details on the gene organization of the subtypes of the α-carboxysome among all sequenced cyanobacterial genomes are also reviewed in Roberts *et al.* 2012 [8].

Alpha- and β-carboxysomes also differ in gene organization. While the core genes of the α-type are organized in an operon (the *cso* operon) (Figure 1), genes of the β-type are located in a conserved locus (the *ccm* cluster) as well as in a few satellite loci [4]. Interestingly, while the β-carboxysome is exclusively found in β-cyanobacteria, the α-carboxysome can be found in not only α-cyanobacteria but also many chemoautotrophs. A *cso* operon was also found in the genome of the eukaryotic alga *Paulinella chromatophora*, a result of a horizontal gene transfer event [5]. *Halothiobacillus neapolitanus* (*Hnea*), a chemoautotroph, has served as a model organism for studying function and structure of the α-carboxysome [6]. Gene organization of *cso* operons from *Hnea*, *Prochlorococcus marinus* str. MED4 (MED4), a high-light adapted strain, and *Prochlorococcus marinus* str. MIT9313 (MIT9313), a low-light adapted strain, are shown in Figure 1. Gene(s) encoding the major shell proteins CsoS1 (containing one Bacterial Microcompartment (BMC) domain, pfam00936) is either the first or last gene(s) of the *cso* operon. The genes *cbbL* and *cbbS* code for the RuBisCO large and small subunits, respectively, followed by genes *csoS2* and a gene encoding a β-class CA, *csoS3*. A pair of paralogous genes, *csoS4A* and *csoS4B,* encode the pentameric vertex proteins (pfam03319) of the carboxysome shell [7]. A gene containing a single BMC domain but with an N-terminal extension (80 to 100 amino acids) of unknown function, *csoS1E*, is unique to α-cyanobacteria, but not found in high-light adapted strains [8]. A gene encoding pterin-4 alpha-carbinolamine dehydratase-like protein is conserved in all known *cso* clusters [4,8,9]. Although it has been proposed as a novel RuBisCO chaperone [9], its absence has no effect on α-carboxysome function as a CO_2_-fixing module in a heterologous host [10]. The product of *csoS1D*, a tandem BMC domain containing gene, has been shown to be a minor component of α-carboxysomes from MED4 [8]. Wildtype and mutant α-carboxysomes can be readily purified to homogeneity from *Hnea*, providing insights on organelle function, protein composition, stoichiometry, and sub-structure localization [6,11,12,13,14]. Furthermore, in the last decade structures of most of the known α-carboxysome proteins were solved [7,15,16,17,18,19]. However, there is an essential piece missing from the model of the α-carboxysome: little is known about function and structure of the product of the *csoS2* gene.

In MED4 and MIT9313, the deduced protein sequence of the *csoS2* gene is 765 and 792 residues, respectively; it is even longer (869 amino acids) in *Hnea* (Table 1). While a single protein corresponding to CsoS2 was identified in purified MED4 and *Thiomicrospira crunogena* carboxysomes [8,20,21], two forms of CsoS2 are found in an approximately 1 to 1 ratio in purified *Hnea* and *Thiomonas intermedia* carboxysomes [20,22]. In *Hnea*, both forms of CsoS2 have intact N-termini [22], which hints at the possibility of C-terminal truncation of the smaller form (CsoS2A). On the other hand, the longer form (CsoS2B) of *Hnea* CsoS2 has an observed molecular weight (MW) of 130 kDa, significantly larger than the calculated MW (92 kDa) [22]. Post-translational modification, such as glycosylation, has been proposed to account for this disparity [22], however, this has not been experimentally verified. In purified carboxysomes, CsoS2 is the third most abundant carboxysome protein following the major CsoS1 shell protein(s) [6,8,20,21]; there are approximately 330 CsoS2 monomers per *Hnea* carboxysome, compared to 270 RuBisCO holoenzymes [6]. Although little is known about the function of CsoS2 other than the fact that it is tightly associated with the carboxysome shell [6,22], this protein may be essential for α-carboxysome function and/or formation.

Mutagenesis approaches have been used to study the formation of the α-carboxysome. Menon *et al.* showed that only the form I RuBisCO that is encoded in the *cso* operon can be encapsulated in the carboxysome, in contrast to the second form I RuBisCO encoded elsewhere in the genome [12]. Furthermore, the large subunit but not the small subunit of form I RuBisCO determines the ability to be encapsulated. In RuBisCO deletion strains, empty carboxysome shells can form without obvious alteration of their size [12].

An electron cryotomography study of *Hnea* cells captured snapshots of semi-assembled α-carboxysomes: structures resembling RuBisCO holoenzyme molecules are lined-up inside of the curvature formed by partial shells [23]. These data suggest that the assembly of the shell and the encapsulation of RuBisCO occur simultaneously in α-carboxysomes. This is distinctly different from the current picture of the biogenesis of the β-carboxysome. Cameron *et al.* [24] showed that the assembly of β-carboxysomes starts from aggregation of RuBisCO with the small subunit-like domains (SSLDs) of CcmM; subsequently, the γ-CA domain of CcmM interacts with CcmN, which has a C-terminal encapsulation peptide (EP) that facilitates the assembly of the shell around this pro-carboxysome core [24]. To date, there are no reports of β-carboxysome shell formation in the absence of RuBisCO.

While CcmM and CcmN are essential for assembling and organizing the interior of β-carboxysomes, there is no homolog to CcmM or CcmN in α-carboxysomes. If the biogenesis of α-carboxysomes is similar to that of β-carboxysomes, a protein to fulfill both the nucleation role of CcmM and the shell-association role of CcmN is required. CsoS2, a protein unique to α-carboxysomes and the only abundant carboxysomal protein with unknown function, is apparently a key protein that perhaps fulfills one or both of these functions. Unfortunately, CsoS2 has proven recalcitrant to crystallization. Therefore, in this study, we combined biochemical, bioinformatic, biophysical, and genetic approaches in an effort to understand its role. We focused this study on CsoS2 from three model organisms: high-light and low-light adapted *Prochlorococcus marinus* str. MED4 or MIT9313, respectively, as well as the genetically tractable model chemoautotroph *Hnea*.

## 2. Results

### 2.1. The Hnea csoS2 Gene Inherently Encodes Two Protein Products

**Figure 2:**
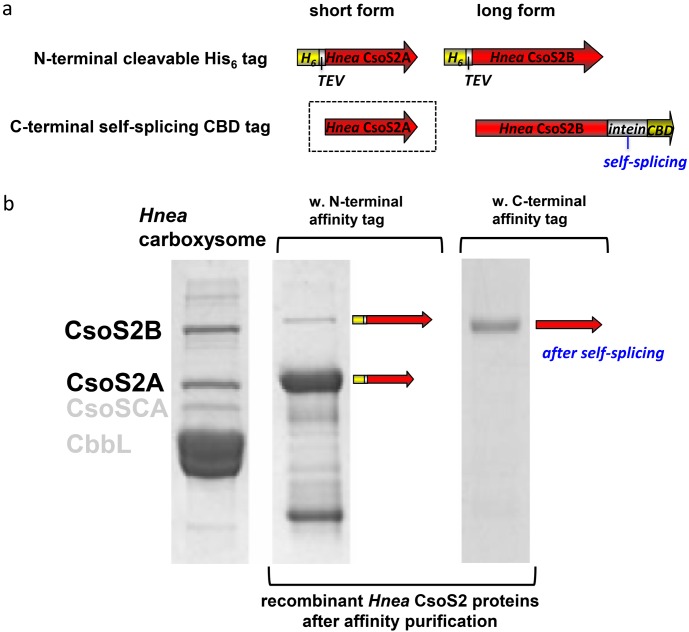
Expression of *Hnea* rCsoS2 in *E. coli* with an N- or a C-terminal tag. (**a**) Schematic of the short and long form of rCsoS2 proteins produced by *E. coli* when codons for either an N- or a C-terminal tag are genetically fused to the open reading frame of *Hnea csoS2* gene. In the case of a C-terminal tag, the short form (boxed by dotted lines) cannot be purified via affinity chromatography because of lack of the C-terminal tag. (**b**) Purified rCsoS2 in comparison with CsoS2A and CsoS2B from native source. The left lane shows the short (CsoS2A) and long (CsoS2B) form of CsoS2 protein in purified *Hnea* carboxysomes. When expressed in *E. coli* with an N-terminal tag, both the short and long forms can be purified using an affinity column (middle lane). When expressed in *E. coli* with a C-terminal tag, only the long form can be recovered after affinity purification followed by self-cleavage of the tag.

To test if the shorter form of *Hnea* CsoS2 is a result of post-translation processing on the C-terminus of the full-length protein, we heterogeneously expressed the *Hnea csoS2* gene in *E. coli* with either an N- or a C-terminal tag. Two forms of the N-terminally tagged CsoS2 can be purified by affinity chromatography, and the short form is more abundant (Figure 2). This may be due to different susceptibilities to proteolysis in *E. coli*, or indicate that most of the N-terminally His-tagged rCsoS2 is expressed as a short form (CsoS2A) with an intact N-terminus. In contrast, only the long form (CsoS2B) was eluted from the affinity resin after self-cleavage of the C-terminal intein tag (Figure 2). Collectively, these findings confirm that CsoS2B is the full-length polypeptide while CsoS2A is C-terminally truncated. The mechanism of truncation is self-contained in the *Hnea csoS2* gene regardless of expression host. In an attempt to identify the C-terminal truncation site, matrix-assisted laser desorption/ionization time-of-light mass spectrometer (MALDI-TOF MS) was used to analyze in-gel Trypsin digested CsoS2A and CsoS2B from purified *Hnea* carboxysomes (Figure S1). The coverage of full-length CsoS2B is 44% and the last detectable peptide covers the sequence up to R868, which is the penultimate residue (Figure S1b). The last detectable peptide of CsoS2A covers the sequence up to R836 (Figure S1a). However, a truncation of N837-G869 will only result in a MW difference of 3.3 kDa; the observed MW difference between CsoS2A and CsoS2B is 45 kDa [6]. Close inspection of the MALDI-TOF results for CsoS2A reveal that there is no coverage over a large region (G574–R826) in contrast to ample coverage in the CsoS2B sample in the same region. Therefore, the coverage between V827–R836 may be an aberration. The last detected residue in CsoS2A prior to this region is R573. A truncated CsoS2 protein with residues 1-573 will yield a calculated MW of 61 kDa, which is 31 kDa smaller than the calculated MW of the full-length CsoS2. This calculated 31 kDa difference is much closer to the observed MW difference of 45 kDa.

### 2.2. A CsoS2 Knockout Mutant Lack carboxysomes

**Figure 3:**
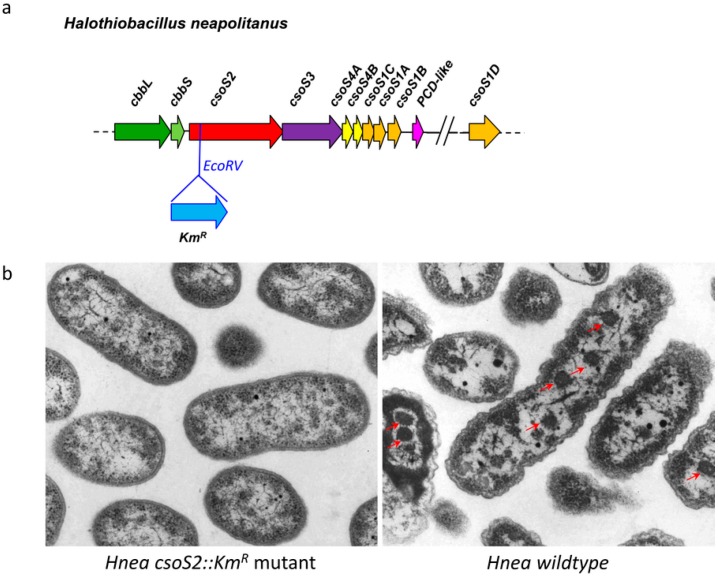
Knockout of CsoS2 abolishes carboxysome formation in *Hnea*. When the *csoS2* gene is interrupted by the insertion of a *Km^R^* cassette (**a**); no carboxysomes are apparent in mutant cells comparing to wildtype *Hnea* cells with carboxysomes (indicated by red arrows) under the same growth condition (**b**).

A *Hnea* *csoS2* gene disruption mutant was generated by inserting a kanamycin resistance cassette (Km^R^) in the *csoS2* coding region at an EcoRV site (Figure 3a). This mutant presents a *high CO_2_ requiring* (*hcr*) phenotype and does not grow in air (Figure S2). This is in contrast to the *hcr* phenotype observed in the *Hnea csoS3* insertion mutant that was similarly constructed, which does grow but at a significantly slower rate than wildtype in air [11]. Thin sections of *Hnea csoS2::Km^R^* mutant cells completely lack carboxysomes (Figure 3b), which accounts for the observed *hcr* phenotype. This is distinctly different from the *Hnea csoS3::Km^R^* mutant, in which the elimination of the CsoSCA protein results in mutant carboxysomes that are indistinguishable in size and appearance from wildtype but functionally defective [11]. The fact that all other carboxysomal proteins are present at a similar level in the CsoSCA knockout mutant relative to wildtype suggests that insertion of a *Km^R^* cassette does not affect expression of downstream genes. These results indicate that the CsoS2 protein is important for the formation or stability of α-carboxysomes.

### 2.3. Hints about the Location of CsoS2 Protein in Hnea carboxysomes

Protein-protein interactions in the *Hnea* carboxysome were screened by yeast-two-hybrid (Y2H) assays [25]. Each carboxysome protein was fused to the DNA binding domain (BD) and the activation domain (AD) of the yeast transcriptional activator protein GAL4 as bait and prey, respectively. The resulting pairwise interaction map further supports an important role of CsoS2 in *Hnea* carboxysome structure (Table S1). CsoS2 is the only protein that interacts with all other carboxysomal proteins as the bait. When fused to the AD (as the prey), CsoS2 strongly interacts with RuBisCO small subunit CbbS, itself, CsoS4B and CsoS1C (Table S1). Although it is known that Y2H assays often have false positive and false negative hits, especially when used to study protein-protein interactions that naturally occur in a prokaryotic system, the strong interaction between CsoS2 and RuBisCO, as well as shell proteins in both directions supports our hypothesis that CsoS2 plays an important role in organizing the interior of *Hnea* carboxysomes. These results also imply that CsoS2 is situated for interaction with both RuBisCO and shell proteins; CsoS2 may be located in between the shell and the lined-up RuBisCO holoenzymes.

### 2.4. SPA-Tagged Hnea carboxysomes Can Be Selectively Purified by Affinity Chromatography

**Figure 4:**
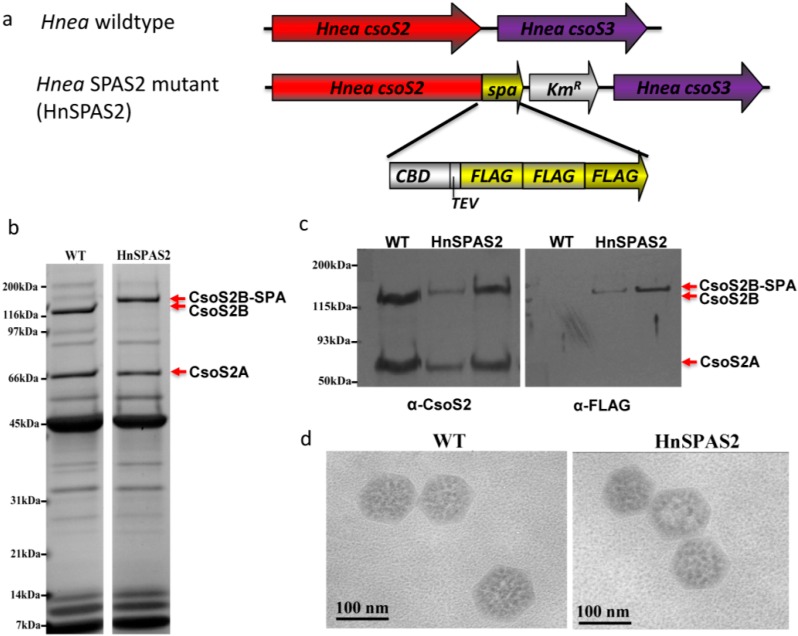
A *Hnea* mutant with SPA-tagged CsoS2. (**a**) The SPA tag fused to the C-terminus of CsoS2 contains a 3x FLAG epitope and a calmodulin binding domain (CBD) separated by a TEV protease site. A kanamycin resistance gene (*Km^R^*) cassette follows the SPA tag to allow for selection. (**b**) Both wildtype and mutant carboxysomes can be purified and their polypeptide separation patterns are similar, except SPA-tagged CsoS2B is slightly larger than untagged CsoS2B. (**c**) Western blots of wildtype and mutant cells blocked against α-CsoS2 antisera and α-FLAG antibodies. Only the long form of CsoS2 has a FLAG epitope tag in *HnSPAS2*. No cross-reactivity with small polypeptides was observed. (**d**) Purified wildtype and *HnSPAS2* mutant carboxysomes are indistinguishable in TEM images.

A sequential peptide affinity (SPA) tag that includes a calmodulin binding domain (CBD), a tobacco etch virus (TEV) protease recognition site and three copies of the FLAG epitope, was fused to the C-terminus of *Hnea* CsoS2 to generate the HnSPAS2 mutant (Figure 4a). This mutant is able to grow in ambient CO_2_, and HnSPAS2 mutant carboxysomes can be purified using the standard protocols [26]. Transmission electron microscopy revealed that the size and shape of HnSPAS2 mutant carboxysomes are indistinguishable from their wildtype counterparts (Figure 4d). The composition of purified HnSPAS2 mutant carboxysomes was analyzed by SDS-PAGE, revealing a similar pattern of polypeptide migration compared to wildtype *Hnea* carboxysomes, with the exception of the C-terminal SPA tagged CsoS2B. The full-length CsoS2 polypeptide migrates more slowly than untagged CsoS2B, as would be expected from the addition of the 7.7 kDa SPA tag (Figure 4b). Cell extracts of wildtype *Hnea* and two HnSPAS2 mutant clones were subjected to immunoblotting (Figure 4c). Probing with α-HneaCsoS2 antisera showed that both short form and long form of CsoS2 are present in the HnSPAS2 mutant, as expected. Probing with α-FLAG antibodies revealed that only the long form contains the FLAG epitope tag. No cross-reactivity of the α-HneaCsoS2 antisera or α-FLAG antibodies with any small polypeptides was observed. These findings confirmed that the long form, CsoS2B, is the full length CsoS2 protein. Interestingly, purified HnSPAS2 mutant carboxysomes can bind to agarose beads conjugated with α-FLAG antibodies and elute with 100 mM glycine buffer, pH 2.5. In contrast, no detectable amount of wildtype carboxysomes is recovered in a comparable pull-down experiment (Figure S3). Densitometric analysis revealed that the mass ratio of polypeptides in the eluted HnSPAS2 carboxysomes is 2:1.5:1:11:1.5:7 for CsoS2B:CsoS2A:CsoSCA:L_8_S_8_RuBisCO:CsoS1B:CsoS1A/C, (L_8_S_8_RuBisCO consists of eight polypeptides each of large (CbbL) and small (CbbS) subunits). These values are similar to those of purified intact HnSPAS2 carboxysomes (CsoS2B:CsoS2A:CsoSCA:L_8_S_8_RuBisCO:CsoS1B:CsoS1A/C = 2.5:2.0:1:13:1:6), suggesting that the trapped carboxysomes were intact.

### 2.5. Unique Primary Structures of Prochlorococcus CsoS2s

**Figure 5:**
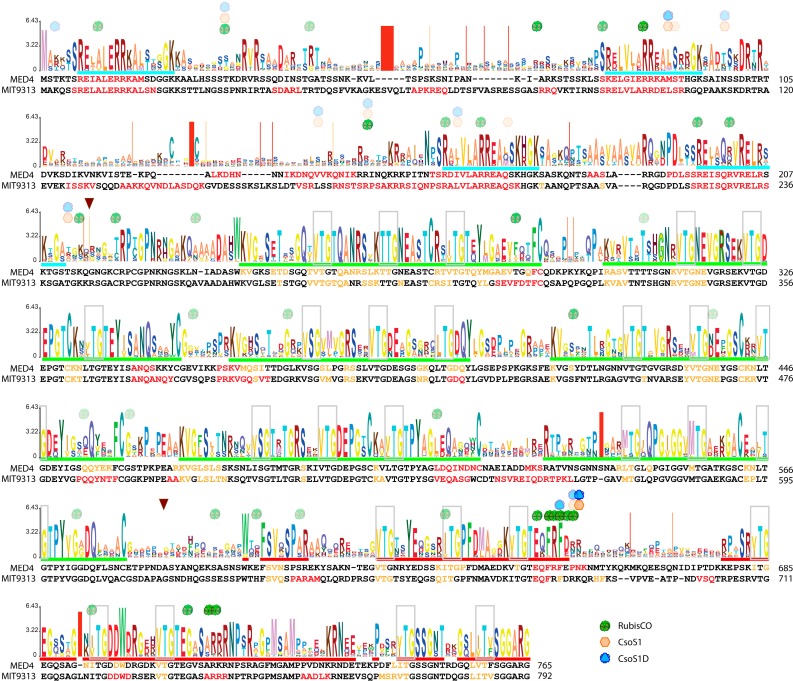
A Hidden Markov model (HMM) logo for all α-Cyanobacterial CsoS2 orthologs. The Y-axis represents the *information content* (aka relative entropy), and the letters divide the stack height according to their estimated probability at a given position. MED4 and MIT9313 CsoS2 sequences are aligned to the corresponding position on the logo, and the predicted secondary structural motifs are colored red and orange for α-helices and β-strands, respectively. N- and M-region repeats are indicated by cyan and green underlining, respectively. Short repeats (3 amino acids) that occur three units per group (except in the last group) are outlined in light-gray boxes. Relatively conserved residues of the C-region are underlined in red. Putative transition areas between three regions are indicated by brown arrows. For demonstration purposes only, a simplified presentation of results from the protein-binding assay against MIT9313 CsoS2 peptide array (see Section 2.12) are mapped onto the logo. The starting position of peptides among all positive hits is marked with RuBisCO, CsoS1 or CsoS1D symbols only if the averaged signal intensity (1) ranks in the top 10 out of all positive hits or (2) is a local maximum with >5 sequential positive hits. The saturation of each symbol is relative to its fraction ratio to the maximum signal intensity (as 100% saturation) of all positive hits from a given binding assay.

Although CsoS2 has the least conserved primary structure among all α-carboxysome proteins [27,28], some unusual sequence features are shared among *Hnea* CsoS2 and its counterparts in *Prochlorococcus* strains. First, the three CsoS2 proteins from *Hnea*, MED4 and MIT9313 have unusually high pI values (Table 1). Secondary structure predictions suggest that CsoS2 can be divided into three regions (Figure 5): an approximately 250 amino acid N-terminal region predicted to be enriched in α-helices and having an even higher pI than the full-length proteins (≥ 1 unit difference); an over 350 amino acid middle (M) region predicted to be predominantly composed of β-strands and having a slightly basic pI (7–8); and a (~170 amino acid) C-terminal region. The pI values of the C-terminal region for MED4, MIT9313 and *Hnea* CsoS2 are 5.26, 7.05, and 9.56, respectively.

**Table 1:** General features found in CsoS2 proteins.

CsoS2 from	N-repeats	M-repeats *	C-region intact ?	length (aa)	calculated pI	Residues Count					
						C	H	R	K	E	Q
*Prochlorococcus marinus* str. MED4	4	6 (TTNTTT)	Y	765	9.63	12	4	43	78	41	31
*Prochlorococcus marinus* str. MIT9313	4	6 (TTNTTT)	Y	792	9.75	12	6	67	44	45	48
*Halothiobacillus neapolitanus* C2	4	6 (TTNTTT)	Y	869	9.06	20	14	63	38	49	51
*Thioalkalivibrio* sp. ALR17-21	5	8 (TTTTTTTT)	Y	1014	7.08	22	22	106	26	87	44
*Thioalkalivibrio nitratireducens* DSM14787	4	6 (TTNTTT)	Y	782	9.19	15	4	78	34	46	34
*Bradyrhizobium* sp. BTAi1	2	5 (SNSNN)	Y	563	10.99	2	8	53	12	20	29
*Thermithiobacillus tepidarius* DSM 3134	4	7 (TTNTNTS)	Y	847	9.90	14	8	79	25	53	37

* M-repeats often contain a signature pattern of cysteine residues near the terminus of the repeat. T, tandem cysteine residues present; S, single cysteine present; N, no cysteine. Y for “yes”.

**Figure 6:**
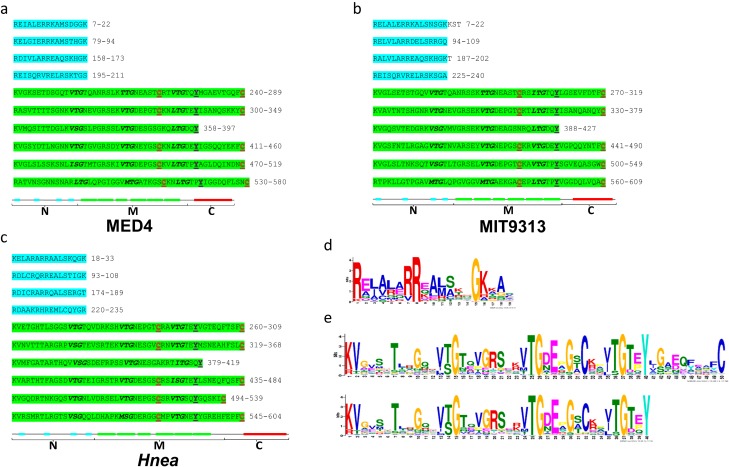
Repetitive motifs found in the three representative CsoS2 proteins. Repetitive motif found in the N-region (cyan) and M-region (green) of MED4 (**a**); MIT9313 (**b**); and *Hnea* (**c**); CsoS2 proteins; (**d**) MEME motif for the N-repeats; (**e**) long and short form of MEME motif for the M-repeats.

One of the distinctive features of the primary structure of CsoS2 is sequence repeats. The M-region consists of six repeats, each composed of three units of three-amino-acid short repeats which are eight amino acids apart from each other (Figure 5 and Figure 6a–c). This observation was first reported for CsoS2 proteins from four Thiobacilli strains, including *Hnea* [27] (Figure 6c). Similar patterns are also evident in MED4 and MIT9313 CsoS2, as shown in Figure 6 (the three-amino-acid short repeats are shown in **bold** and ***italic***). In each case, by aligning all six repeats, other features emerge. For example, all M-region repetitive patterns (M-repeat) are approximately 50 amino acids long except the third one, which is 10 amino acids shorter. Some well-conserved residues stand out: a tyrosine and a pair of cysteine residues in all except in the shorter forms.

Interestingly, a close examination of the primary structure of the N-region reveals another repetitive motif (N-repeat), which is different from the M-repeat motif (Figure 5 and Figure 6). This motif is 16 amino acids in length, begins with a basic residue (R or K), and is followed by an acidic residue (E or D, in all but one case). For CsoS2 from MED4 or MIT9313, these 16 amino acid segments are predicted to form α-helices (Figure 5).

### 2.6. The Three-Region Architecture of CsoS2 Is Conserved

A comprehensive survey of CsoS2 orthologs from diverse bacterial genomes indicates that the three-region architecture, as well as the repetitive arrangements in both N- and M-regions, occurs ubiquitously. This survey included all currently available CsoS2 sequences (165 sequences found in the Integrated Microbial Genomes database, https://img.jgi.doe.gov as of September 2014; Table S2). Thirty-seven sequences are from cyanobacteria of which 35 are marine *Synechococcus* and *Prochlorococcus*; the remaining 128 are from chemoautotrophs belonging to the phyla Actinobacteria, Nitropirae and Proteobacteria, including some nitrifying or sulfur-oxidizing bacteria as well as some purple phototrophs. These predicted CsoS2 gene products have MW values ranging from 56.96 kDa (563 aa; *Bradyrhizobium* sp. BTAi1) to 107.61 kDa (1019 aa; *Thioalkalivibrio* sp. ALE19) and pI values ranging from 7.08 (*Thioalkalivibrio* species ALE20, ALE19 and ALR17-21) to 11.3 (*Marichromatium purpuratum* 984). Representatives are listed in Table 1. Interestingly, although not all CsoS2 proteins have an extremely high pI, the ubiquity of three-region architecture and the repetitive arrangements in both N- and M-regions is consistent for all cases. The C-region appears to be relatively more conserved than the full-length protein (Table 2), while the number of repeating motifs in the N- or M-region varies and correlates with the relative the length of each protein (Table 1). Notably in the M-repeats, cysteine residues do not always occur as a pair; a single cysteine can be found (Table 1). A Hidden Markov model (HMM) logo for all unique CsoS2 orthologs from α-cyanobacteria is shown in Figure 5. The third M-repeat seems to be a short form in all cyanobacterial CsoS2s. The three-amino-acid short repeats found within the M-repeat are also present in the C-region of CsoS2 (Figure 5). We identified a 19 amino acid-long motif with an extraordinarily low E-score (2.4e^−2099^) for the N-repeat (Figure 6d). Two motifs with different lengths were identified for the M-repeat (Figure 6e), each corresponding to the long and short form described above (Figure 6a–c). They are 50 and 40 amino acids long with an E-score of 4.3e^−11,260^ and 1.8e^−10,176^, respectively.

**Table 2:** Pairwise alignment matrix of C-region comparing to that of full-length CsoS2.

	MED4	MIT9313	*Hnea*	ALR17-21	*Tni*	*Brady*
**C-region: I/S**						
MED4						
MIT9313	58.5/73.3					
Hnea	34.7/45.3	37.3/49.4				
ALR17-21	38.1/49.7	**43.2**/**54.7**	42.5/56.9			
Tni	**51.7**/60.7	**58.2**/68.8	**40.7**/49.4	**44.0**/**56.0**		
Brady	**34.3**/**47.1**	**34.8**/**45.9**	26.9/37.5	**30.8**/**41.8**	**37.6**/45.4	
Tte	**38.6**/**54.3**	**44.8**/**60.4**	41.4/56.1	43.8/**57.6**	**45.1**/53.5	**35.1**/**47.0**
**full-length: I/S**						
MED4						
MIT9313	54.1/69.5					
Hnea	30.8/45.6	32.3/46.4				
ALR17-21	29.2/42.0	**29.2**/**40.3**	33.1/47.3			
Tni	**41.2**/56.4	**45.0**/59.5	**29.7**/39.5	**32.1**/**43.1**		
Brady	**22.8**/**32.8**	**22.1**/**31.4**	20.6/30.6	**19.8**/**27.5**	**26.2**/35.6	
Tte	**28.7**/**42.7**	**31.6**/**43.7**	41.7/54.7	35.8/**47.6**	**34.4**/44.1	**22.6**/**32.7**

* More than 10% difference in identity (I) or similarity (S) value is denoted by **bold**. ALR17-21, *Thioalkalivibrio* sp. ALR17-21; *Tni*, *Thioalkalivibrio nitratireducens* DSM 14787; *Brady*, *Bradyrhizobium* sp. BTAi1.

### 2.7. Phylogenetic Analysis of CsoS2 Hints at the Origin of α-carboxysomes

**Figure 7:**
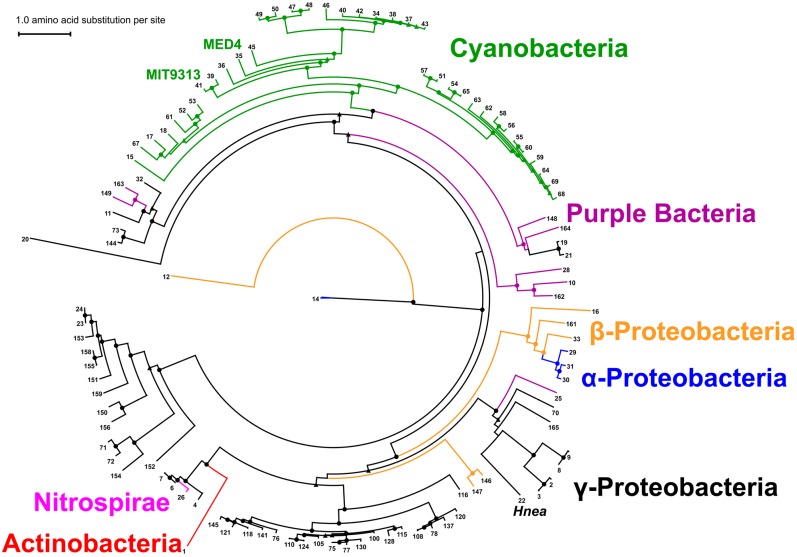
A CsoS2 phylogram. CsoS2 orthologs (Table S2) found in α-Cyanobacteria (green), α-Proteobacteria (blue), β-Proteobacteria (orange), γ-Proteobacteria (black), Actinobacteria (red) and Nitrospirae (magenta) are shown in the phylogram. Purple phototrophs, which belong to the γ-Proteobacteria, are shown in purple. Bootstrap values were obtained from 100 replicates; nodes receiving bootstrap values greater than 75 or between 50 and 74 are indicated by filled circles or filled triangles, respectively. Numbers correspond to organism ID numbers given in Table S2.

A phylogenetic tree based on the maximum-likelihood method with 100 Bootstrap (*bs*) replicates was constructed using all CsoS2 orthologs. CsoS2 orthologs from the Cyanobacteria form a single clade (*bs* = 90%), while CsoS2s from purple phototrophs form at least two clades (*bs* = 90% and 50%), one being a sister clade of the Cyanobacterial CsoS2. The topology of the CsoS2 tree is different from that of the bacterial phyla tree [29], and it suggests that CsoS2 may have first occurred in the common ancestor of the Proteobacteria (Figure 7). The possibility that α-cyanobacteria obtained α-carboxysomes via a horizontal gene transfer (HGT) event from Proteobacteria has been proposed [30,31]. This hypothesis is also consistent with the absence of either α- or β-carboxysomal genes in bacterial genomes belonging to Melainabacteria, a sibling phylum sharing a common ancestor with both α- and β-cyanobacteria [32]. Possible HGT events are also evident in the cases of α-carboxysome containing genomes belonging to Actinobacteria and Nitrospiare (namely, *Acidimicrobium ferrooxidans* DSM 10331 and *Leptospirillum ferriphilum* BYQ, respectively) (Figure 7).

### 2.8. Structure Prediction of CsoS2: An Intrinsically Disordered Protein?

**Figure 8:**
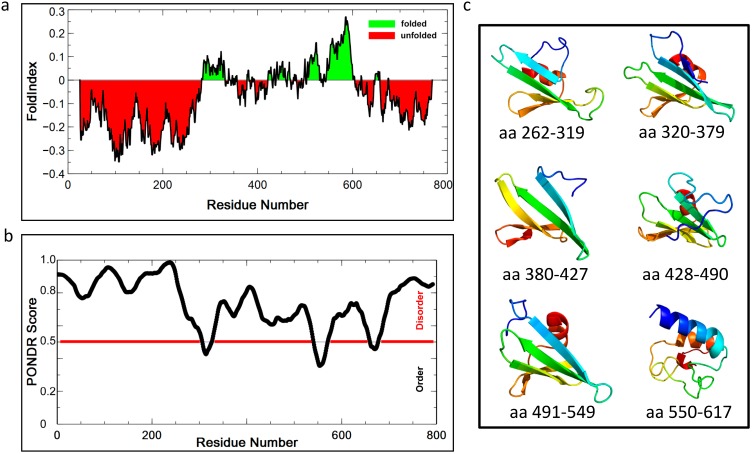
Folding predictions for MIT9313 CsoS2. (**a**) Fold-Index prediction; (**b**) PONDR prediction; (**c**) ribbon presentation of *ab initio* folding prediction by QUARK for each M-repeat, shown in a rainbow spectrum from N-terminus (blue) to C-terminus (red).

Protein Basic Local Alignment Search Tool (BLASTp) searches using the primary structures of *Hnea*, MED4 or MIT9313 CsoS2 against Protein Data Bank (PDB) with an E-value cut-off of 1.0 returned no hits. Therefore, we undertook *in silico* tertiary structure prediction for all available CsoS2 sequences. The predictions resulting from two different algorithms are similar and the majority of sampled CsoS2 orthologs are predicted to be disordered. Only a few exceptions have more than one region (>50 residues) predicted to adopt a local fold (CsoS2 from *Acidimicrobium ferrooxidans* DSM 10331, *Bradyrhizobium* sp. BTAi1 and *Thermithiobacillus tepidarius* DSM 3134). Predictions for the MIT9313 CsoS2 are shown in Figure 8a,b as examples. Using FoldIndex, more than 70% of the sequence is predicted as unfolded (in red) with three locally folded regions (in green) (Figure 8a), each of which roughly corresponds to one of the three big dips in the PONDR prediction (Figure 8b), indicating potential order locally or order formed when a binding partner is present. The first is located in the first M-repeat; the second occurs between the 5th and 6th M-repeats, and the last is at the beginning of the C-region. The consistency in prediction of locally-ordered regions suggests the possibility of the M-region adopting a beads-on-a-string conformation, with beads representing local units of tertiary structure. We performed *ab initio* protein structure prediction for each M-repeat with flanking sequences. Excluding the sixth repeat, four out of five long M-repeats all adopt a [(β-strand)_4-6_-(α-helix)_1-2_] conformation (Figure 8c). The third M-repeat (short form) also adopts a (β-strand)_4-6_ conformation but lacks the α-helix portion due to the absence of 10 amino acid C-terminal extension (Figure 6d). Together, these results suggest that CsoS2 is highly flexible, and a beads-on-a-string conformation is possible at least for the M-region.

### 2.9. Experimental Data Supports the Prediction of a Highly Flexible CsoS2

**Figure 9:**
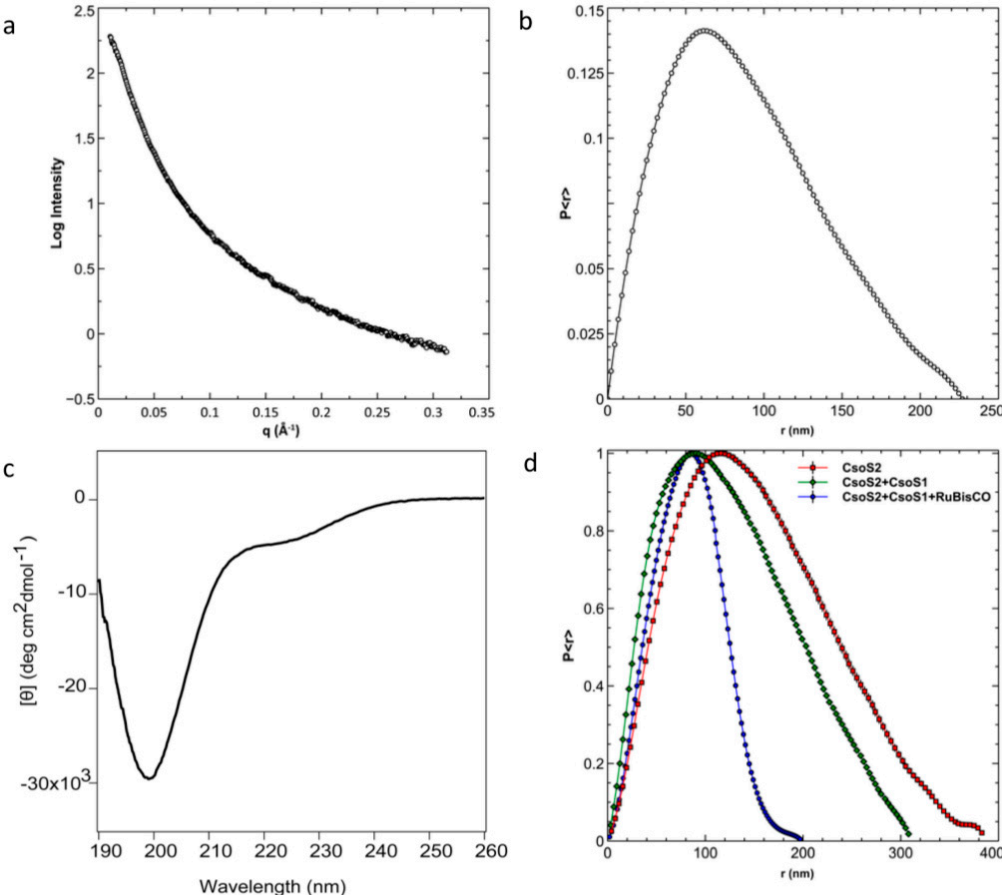
Biophysical characterizations of *Prochlorococcus* CsoS2. (**a**) SAXS analysis of MED4 CsoS2 and (**b**) the pair distribution function of the same SAXS data; (**c**) Near UV Circular Dichroism spectroscopy of the same protein; (**d**) The pair distribution functions of SAXS data measured on MIT9313 CsoS2 in isolation and after mixing with MIT9313 CsoS1 and MIT9313 RuBisCO.

To characterize *Prochlorococcus* CsoS2, we expressed the MED4 and MIT9313 CsoS2 orthologs as full-length, as individual N-, M- or C-regions and as N- or C truncation (MC or NM, respectively) with a cleavable glutathione S-transferase (GST) tag fused to the N-terminus. Taking advantage of tandem purification (cation exchange chromatography followed by affinity chromatography) and on-column GST-tag cleavage, full-length or shorter versions of CsoS2 proteins were purified and concentrated to approximately 30 mg/mL in 20 mM HEPES pH 7.4 with 50 mM NaCl. Full-length MED4 CsoS2 was subjected to Small Angle X-ray Scattering (SAXS). The SAXS curve has an overall smooth shape without a local minimum or maximum (Figure 9a). Similar observations have also been reported for other highly flexible proteins, and smoothness is considered one of the characteristic features of SAXS data for intrinsically flexible proteins [33,34]. Calculation of the pair distribution function, *P<r>*, shows a shape compatible with an elongated conformation, with a radius of gyration of 69.3 ± 0.26 nm and a maximum diameter of 226 nm (Figure 9b), which does not support a “beads-on-a-string” structure under the testing (solution) conditions with protein concentrations up to 8.5 mg/mL. MED4 CsoS2 was also subjected to Circular Dichroism (CD) spectroscopy (Figure 9c). The dominant single minimum at 199 nm indicates a high percentage of random coil conformation [35], which is consistent with the structural prediction and the SAXS observations. Size exclusion chromatography (SEC) of MED4 CsoS2 results in an observed MW of ~250 kDa, approximately three times that of the theoretical monomer MW (82 kDa). However, the estimated MW by SEC is based on the assumption that the protein is globular, and considering that the predictions for disorder in CsoS2 this estimate may be spurious.

### 2.10. CsoS2 Interacts with the Major Shell Protein CsoS1 and RuBisCO in vitro

Given the evidence that CsoS2 is a carboxysome shell-associated protein [6,22,26], we probed for interactions between CsoS2 and shell proteins *in vitro*. Purified MED4 CsoS2 (~6 mg/mL) was mixed with purified MED4 CsoS1 at molar ratios from 16:1 to 1:64, which spans the reported molar ratios of CsoS2 to CsoS1 in purified α-carboxysomes from different organisms [1,6,8]. An increase in opacity from the initial transparent solution was observed immediately. A plot of ΔOD_600_, as an estimate of turbidity, against CsoS1:CsoS2 molar ratio is shown in Figure S4, there is a major peak between 1:1 and 1:10 ratios. Purified full-length or shorter versions of MIT9313 CsoS2 (~7 mg/mL) were also mixed with the same volume of MIT9313 CsoS1 (~15 mg/mL) at pH 7.4. For full-length CsoS2, CsoS2-N and CsoS2-NM, precipitates were immediately observed; however, when CsoS2-M, CsoS2-C and CsoS2-MC were mixed with CsoS1, protein samples remained clear. This suggests the N-region of CsoS2, which has the highest pI among the three regions, is responsible for the observed increase in turbidity after mixing. However, in addition to CsoS2-N, both CsoS2-NM and full-length CsoS2 also carry net positive charges under the testing conditions. This suggests that protein precipitation upon mixing may not be dependent on charge. To test this, commercially available proteins with high pIs (ranging from 8.2–10.0; prepared at 10 mg/mL) were each mixed with MIT9313 CsoS1. None of the mixtures precipitated, suggesting the observed precipitation is indicative of relatively specific protein-protein interactions between these two carboxysome proteins.

Based on the tendency to elicit precipitation, CsoS2 was also tested for interaction with RuBisCO. MIT9313 CsoS2 and CsoS1 were mixed, followed by addition of recombinant MIT9313 RuBisCO. The mixture was analyzed by SAXS. Comparing the distance distribution function, *P<r>*, both the radius of gyration and the maximum diameter of CsoS2 decrease significantly upon addition of CsoS1 and then RuBisCO (Figure 9d), suggesting a transition of CsoS2 from an elongated shape to more compact conformation in the presence of the other two carboxysome proteins.

**Figure 10:**
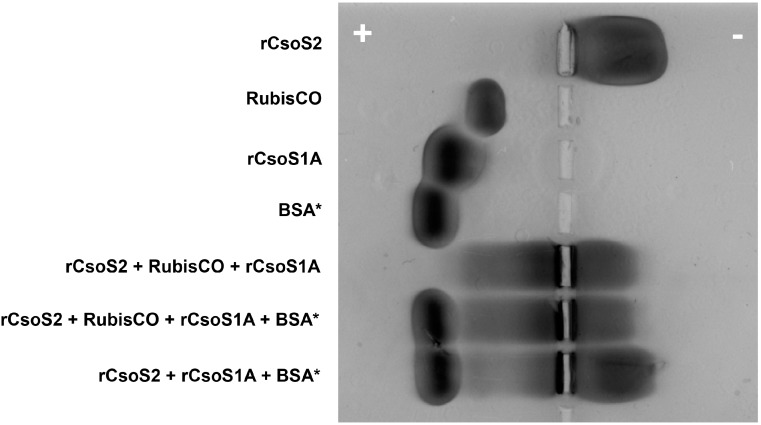
Native agarose gel electrophoresis of *Hnea* recombinant carboxysome protein and *Hnea* RuBisCO mixtures. Lanes from top to bottom: rCsoS2 (20 µL at 0.6 mg/mL), RuBisCO (20 µL at 1.1 mg/mL), rCsoS1A (20 µL at 0.7 mg/mL), BSA (20 µL at 1.0 mg/mL), rCsoS2 w. RuBisCO and rCsoS1A (20 µL each), rCsoS2 w. RuBisCO, rCsoS1A and BSA (20 µL each), and rCsoS2 w. BSA and rCsoS1A (20 µL each). By itself, the positively charged rCsoS2 migrates to the negative electrode; rCsoS1A and RuBisCO migrate to the positive electrode. When mixed, rCsoS2 drags its interaction partners, but not BSA, towards the negative electrode.

Similarly, protein-protein interactions were observed upon mixing of soluble recombinant *Hnea* CsoS2, the shell protein CsoS1A, and *Hnea* RuBisCO purified from the native source. In native agarose gels, the positively charged rCsoS2 migrates to the negative electrode; rCsoS1A and RuBisCO migrate to positive electrode (Figure 10). When mixed, all of the proteins, presumably as a complex, migrate to the negative electrode. Commercially available BSA was used as a control and it only migrates to the positive electrode in the absence or presence of CsoS2 in native agarose gels (Figure 10), which further supports the hypothesis that protein-protein interactions between CsoS2 and shell proteins or RuBisCO are specific.

### 2.11. In vivo Formation of CsoS2-CsoS1 Protein Complexes in the Presence of CsoS1D in E. coli

**Figure 11:**
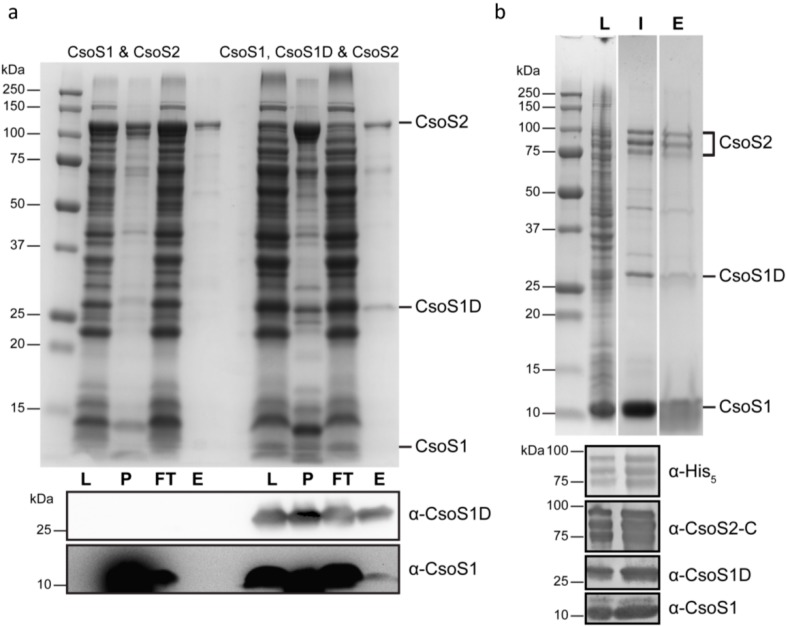
Pull-down assay and purification of α-carboxysome interaction complexes. (**a**) SDS-PAGE and Western blots (α-CsoS1 and α-CsoS1D) of lysate (L) from cells co-expressing CsoS1 and CsoS2 or CsoS1, CsoS1D and CsoS2 and the corresponding cell debris after breaking the cells (P), flow-through fractions (FT) and elutions (E) after pull-down assay using Glutathione-Sepharose magnetic beads; (**b**) SDS-PAGE of lysate (L) from cells co-expressing CsoS1, CsoS1D and CsoS2 from a single construct, the isolated complex (I) and the elution (E) from pull-down assay using Ni-NTA-agarose magnetic beads. Western blots for samples L and I are shown underneath using four different antibodies: α-His_5_, α-CsoS2-C, α-CsoS1 and α-CsoS1D. All three bands that have higher MW (between 75–100 kDa) are identified as CsoS2 with intact C-termini.

The protein precipitates frequently formed by rCsoS2 and rCsoS2 upon interaction with other carboxysome components *in vitro* in protein-protein interaction studies make it difficult to isolate complexes of defined stoichiometry. Therefore, co-expression of potential binding partners and isolation of interaction complexes were pursued. GST-MED4 CsoS2 (pFC005) was co-expressed with CsoS1 alone (pFC117) or CsoS1 and CsoS1D (pFC119) (vector information see Table S3). Pull-down experiments were performed for both. Although no CsoS1 was found in elution fractions examined by coomassie blue stained SDS-PAGE, when CsoS1D is also co-expressed, CsoS1 can be detected in the elution fraction by Western Blotting (Figure 11a). CsoS1D is present at approximately a 5:1 molar ratio to GST-CsoS2 based on densitometry of stained SDS-PAGE (Figure 11a, fraction E). In contrast, the estimated copy number of CsoS1D:CsoS2 is 1:4 based on analysis of purified MED4 carboxysomes [8]. Since stoichiometry is likely a factor for proper complex assembly, we next varied protein expression levels using *E. coli* RBS with different strengths. A single-plasmid co-expression construct, pFC215, was built with codon-optimized *csoS1*, *csoS1D* and *csoS2* genes and artificial intergenic regions. CsoS1-CsoS1D-CsoS2 complexes were purified following a protocol modified and adapted from both α-carboxysome purification and synthetic rBMC shell purification protocols [2,8]. Interestingly, all three high MW polypeptides present in the purified sample are identified as CsoS2 with intact C-termini (Figure 11b). Pull-down experiments were also performed for this expression construct, and the protein composition of the pulled-down fraction is similar to that of purified complex (Figure 11b).

### 2.12. Exploring the CsoS2 Protein Interaction Network Using Peptide Arrays

Taking into consideration the flexibility of CsoS2 and its interaction with carboxysome shell proteins as well as with RuBisCO, we further probed its interaction network using peptide arrays. We used a library of overlapping peptides (8-mers), each shifted by one amino acid to span the entire sequence of MIT9313 CsoS2. Each peptide has at least four replicate spots per library; three identical libraries were each designated for one specific carboxysome protein in a given binding assay: RuBisCO holoenzyme, CsoS1 and CsoS1D. Raw data and average signal values with their standard deviations and p-values are given in Table S4. A simplified presentation of the resulting peptide-binding specificities is mapped onto the MIT9313 CsoS2 sequence and the HMM logo of CsoS2 (Figure 5). The starting positions of the peptides are marked with RuBisCO, CsoS1 or CsoS1D symbols only if the averaged signal intensity (1) ranks in the top 10 out of all positive hits in a given binding assay or (2) is a local maximum with >5 sequential positive hits. The opacity of each symbol is relative to its percentage ratio to the maximum signal intensity (as 100% saturation) of all positive hits from the corresponding binding assay. Positive hits for RuBisCO holoenzyme are observed in all three regions of CsoS2; the binding patterns of CsoS1 and CsoS1D are similar with the exception of the M-region. A region spanning 23 amino acids (ranging from I673 to A695), located in the relatively conserved C-terminal region of CsoS2 (Figure 5), is evidently a hot-spot for protein-protein interactions between CsoS2 and other carboxysome proteins (Figure 5). Notably, the highest signal in each binding assay is observed in this region (Table S5). Six out of the ten peptides with top signal intensities fall in this range for RuBisCO, and this number is two and three for CsoS1 and CsoS1D, respectively (Table S5). In the case of RuBisCO, peptide #681 has the highest signal while its neighboring peptides #679, #680, #682 and #683 also have comparably high signal intensity (Tables S3 and S4). This indicates a large interaction site/pocket for RuBisCO. On the other hand, peptide #683 has the highest signal for CsoS1 and CsoS1D, shifting 2 amino acids from that of RuBisCO (Figure 5). This stand-alone position suggests that CsoS1 and CsoS1D interact with CsoS2 at this position within a much narrower site/pocket.

In all three cases, binding is also observed in the N-region of CsoS2, mostly where N-repeats are located (Figure 5). Interactions in the M-region are only observed for RuBisCO. It appears that RuBisCO tends to bind at the beginning or the end of M-repeats (Figure 5). The positive charge of M-repeats might facilitate such interaction since the RuBisCO of α-carboxysomes is more negatively charged than that of β-carboxysomes and plants [36]. In addition to the 23 amino acid region (I673 to A695) mentioned above, another significant interaction region for RuBisCO is found ranging from E735 to A756; peptide #739 has a signal intensity 86% of the maximum observed in the RuBisCO binding assay (Table S4). This segment also shows some binding affinities for CsoS1 and CsoS1D, with signal strength approximately 1/10 that of RuBisCO (Table S4).

## 3. Discussion

Understanding the biogenesis of carboxysomes is of fundamental importance for understanding the impact of compartmentalization on CO_2_ fixation as well as for prospects for engineering architecturally similar bacterial organelles and for their transfer into heterologous systems (e.g., plants) [37]. Whether or not α-carboxysomes follow an assembly pathway similar to that of β-carboxysomes is an open question.

Alpha-carboxysomes are consistently smaller in size with a thinner shell compared to β-carboxysomes. Reported average diameters for α-carboxysomes vary from 100 to 134 nm; the observed diameters for β-carboxysomes vary from 200 to 400 nm [19,38,39,40,41]. Small variations are also noted between individual carboxysomes from a single species and can arise from differences in sample preparation or imaging techniques [23,30,42,43,44,45]. The thickness of carboxysomal shells is 3–4 nm and 5–6 nm for α and β, respectively [43,44,46]. Although the α- and β-carboxysomes play the same functional role in the CCM, they each form a monophyletic group, without a sister relationship, when compared to other structurally related but functionally distinct bacterial microcompartments [31]. Rae at al. proposed a convergent evolutionary relationship between α- and β-carboxysomes [40]. If this hypothesis is correct, assembly of α- and β-carboxysomes may be fundamentally different. Recently, a step-wise pathway, proceeding from core formation to encapsulation by the shell was proposed for the biogenesis of β-carboxysomes [24]. It has recently been proposed that the majority of functionally distinct types of BMCs assemble by forming the metabolic core, followed by encapsulation by the shell [47].

Our study of the assembly of and protein-protein interactions in α-carboxysomes was mainly carried out in the model system *Hnea*, taking advantage of its tractable genetics and the relative ease of purification of carboxysomes from this organism, in contrast to cyanobacteria. Physical disruption of purified α-carboxysomes results in free RuBisCO and a separate shell fraction, which includes CsoS1A/B/C, CsoS2, CsoSCA and CsoS4A/B [6,11,22]. These shell components can be further categorized as major (CsoS1A/B/C and CsoS2) and minor components (CsoSCA and CsoS4A/B) based on stoichiometry [6]. It seems that the absence of the minor components has no or little effect on biogenesis of carboxysomes but does affect carboxysome function [11,14]. The structural role of CsoS1A/B/C, the major shell components, in carboxysome biogenesis is quite obvious in light of crystallographic studies: these hexameric proteins tile together as basic building blocks for a single layer forming the facets of carboxysome shell [7,17,18]. However, knowledge of the role of CsoS2, the largest carboxysomal protein and the only other major shell component, is limited.

As we show in this study, absence of CsoS2 completely abolishes carboxysome formation, which implicates CsoS2 as a critical component in biogenesis of α-carboxysomes. Results from an *in vivo* Y2H screen of *Hnea* carboxysomal proteins and *in vitro* biophysical characterization of interactions between MED4 or *Hnea* shell proteins with RuBisCO suggest CsoS2 mediates protein-protein interactions between RuBisCO and the carboxysome shell. Interestingly, empty carboxysomes can be assembled *in vivo* in the absence of carboxysomal RuBisCO [12]; this presumably would not be possible if α-carboxysome assembly is similar to β-carboxysome assembly, which requires RuBisCO aggregation as the initial step [24]. Indeed, partially assembled α-carboxysomes without lumen contents were observed in an electron cryotomographic study [23]. Furthermore, it is the large but not the small subunit of form I RuBisCO that determines whether the protein can be sequestered into α-carboxysomes [12]. Collectively, these results point to an important role for CsoS2 in the biogenesis of α-carboxysomes.

Our study systematically dissects sequence and structural features likely to be critical to the role of CsoS2 in the α-carboxysome. CsoS2 can be parsed into three regions, and we identified repetitive motifs in both the N- and M-regions. We propose that these unique primary structural features of CsoS2 are essential to its function(s). While the C-region is relatively consistent in length across species, the number of N- and M-repeats varies, largely depending on species. This observation also suggests different functional roles for the N-, M- and C-regions. Although the mechanism by which the *csoS2* gene codes for one or two polypeptides is not fully understood [8,20,21,22], we present multiple lines of evidence to support our proposal that *Hnea* CsoS2B is a full-length product and *Hnea* CsoS2A has an intact N-terminus but a shortened C-terminus. This feature is reminiscent of the long and short forms of CcmM in β-carboxysomes [48], and this observation is considered in our proposed model for CsoS2 (discussed below).

Both CsoS2 and CsoSCA are carboxysome shell-associated proteins [6,11,22]. While CsoSCA is proposed to bind to the inside of the shell [11], possibly through its carboxysome-specific N-terminal domain [16], little is known about how CsoS2 interacts with the carboxysome shell spatially. There are three possibilities: (1) CsoS2 binds to the shell on its interior and is only accessible from the lumen; (2) CsoS2 binds to the shell on its exterior; and (3) CsoS2 is integrated into the facets of the carboxysome shell, resulting in partial exposure to both lumen and cytoplasm. The first possibility can be ruled out due to some experimental observations. When a SPA tag is C-terminally fused to *Hnea* CsoS2 protein, mutant but not wildtype carboxysomes selectively bind to an affinity column. This suggests that at least the C-terminus of full-length CsoS2 is exposed on the outside of intact carboxysomes. Furthermore, only CsoS2 proteins (both long and short forms) can be instantly and heavily labeled in a crosslinking experiment of intact *Hnea* carboxysomes using a primary amine-active reagent conjugated with biotin [49]. This also suggests at least part of the CsoS2 polypeptide is exposed to the outside. These two observations can also be explained by a scenario where CsoS2 is present in two populations, one binding to the shell on its interior, one binding the exterior (cytoplasmic) surface. This alternative explanation seems less likely because purified carboxysomes (both intact and after disruption) do not appear to have RuBisCO molecules, which tightly interact with CsoS2, adhering to the outer surface.

**Figure 12:**
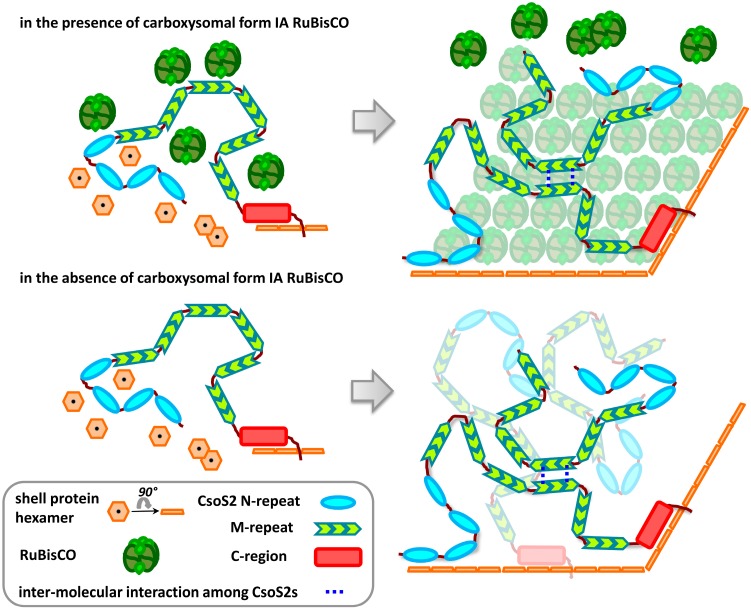
A working model for the location and function of CsoS2 in α-carboxysome assembly. Prior to α-carboxysome formation, RuBisCO and shell proteins such as CsoS1 are recruited by CsoS2. Subsequently, CsoS1 hexamers tile together and form shells anchored by CsoS2 via its C-region, and RuBisCO line up while associated with CsoS2. As a result, the carboxysome is assembled during the simultaneous formation of the shell and packing of RuBisCOs. CsoS2 may adapt different conformations in the final stage; a network of CsoS2 is formed based on inter-molecular interactions among CsoS2 proteins, which may be mediated through disulfide bonds formed between conserved Cysteine residues found in M-repeats. Short forms of CsoS2 (CsoS2A) will only organize RuBisCO but not provide anchoring to the shell. The tail of C-region may be exposed on the surface of the carboxysome and accessible from cytoplasm.

The results of our study suggest CsoS2 is highly flexible and that its three distinct regions have different binding specificities for RuBisCO and shell proteins. From these data and the results of prior studies, we propose a model for CsoS2 function and spatial location in α-carboxysomes (Figure 12). First, the N-region of CsoS2 recruits CsoS1 shell protein(s); when the local concentration increases to a threshold amount, CsoS1 starts to self-assemble into a single layer anchored upon the C-region of CsoS2 but leaving the tail of the C-region exposed to the cytoplasm. Simultaneously, RuBisCO coalesces with CsoS2 through protein-protein interactions, and a lattice of RuBisCO starts to form around the M-region while also simultaneously anchored to the C-region of CsoS2. As a result, RuBisCO is organized by the M-region of CsoS2. A network of CsoS2 is formed based on inter-molecular interactions among CsoS2 proteins, which may be mediated through disulfide bonds formed between conserved cysteine residues found in M-repeats. Carboxysomes of some species also have a short form of CsoS2, which is composed of only N- and M-regions. This form of CsoS2 would not be in contact with the shell but would be expected to organize the inner layers of RuBisCO only. In the absence of carboxysomal form IA RuBisCO, empty shells form, which contain both long and short form of CsoS2 due to the protein-protein interaction among CsoS2 proteins. This model is consistent with the majority of experimental observations. For example, in this model, only the outermost layer of RuBisCO can bind to the C-region of CsoS2 since CsoS2 is anchored on the shell via its C-region; RuBisCO of the inner layers interacts less strongly with flexible CsoS2 through the M-regions. This would explain the observation that the outmost layer of RuBisCO, which is closest to the shell, is the most ordered, while inner layers of RuBisCO may be a result of more random packing [44,45]. The model is also supported by the approximately 1/3 of carboxysomal RuBisCO that remains with the shell fraction when *Hnea* carboxysomes are disrupted by freeze-thawing; this RuBisCO cannot be released from the shell [50]. Although CsoS2 adopting a beads-on-a-string conformation for the M-region is not supported by solution state data on isolated CsoS2, it may still be the case *in vivo*; when it is packed with its interaction partner RuBisCO in a micro-environment where the local protein concentration is extremely high (approximately 900 mg/mL; see supporting methods for calculation). Furthermore, CsoS2 is predicted to be an intrinsically disordered protein (IDP) (Section 2.8, above); IDPs frequently adopt local folds specifically in the presence of interaction partners [51,52,53].

Despite the differences in their constituent proteins, analogies can be drawn between our model for α-carboxysome assembly to the β-carboxysome assembly pathway. While the anchoring role of the C-region could be a functional counterpart of EP in β-carboxysomes [54], the M-region with repetitive units functioning as a locus of aggregation mirrors the role of CcmM, with its species-dependent 3–5 units of SSLD [36], in organizing RuBisCOs in β-carboxysomes. Interestingly, knowing that M-repeats of CsoS2 are positively charged, a survey of total 332 SSLDs from 94 CcmM sequences reveals that 92.5% of SSLDs have a calculated pI over 7. Furthermore, the SSLDs of CcmM presumably adopt the α/β/α sandwich fold of the RuBisCO small subunit; the M-repeat of CsoS2 is also predicted to adopt an α/β/α sandwich fold. We suggest that the interaction between CsoS2 and RuBisCO in the α-carboxysome is analogous to the interaction between CcmM and RuBisCO in the β-carboxysomes [24,55]. Notably, CcmM of *Synechococcus elongatus* PCC7942 has a long form (M58, with both γ-CA domain and SSLDs) and a short form (M38, SSLDs only); *Hnea* CsoS2 also has a long form (full-length) and a short form (comprised of only the N- and M-regions). These coincidences may be taken as evidence for similar functions for CsoS2 and CcmM.

Finally, in the last decade the concept of intrinsically disordered proteins (IDP) has emerged and has drawn considerable attention among researchers in diverse biological systems [51,52,56]. Our data suggests that CsoS2 may be an IDP. IDPs often have the ability to change conformation upon binding to their partner, such as other proteins or small ligands [52,53,57]. Their flexible nature is the key to their unique functions. Our model for α-carboxysome assembly does not preclude intrinsic disorder for isolated CsoS2, with local folding only in the presence of other carboxysome proteins. Given that many IDPs function as scaffolds and interact with numerous other proteins [51,58,59,60], intrinsic disorder may be a requisite property of CsoS2 facilitating its role as a scaffold for α-carboxysome formation.

## 4. Materials and Methods

### 4.1. Sequence Analysis and Bioinformatics

All CsoS2 amino acid sequences were retrieved from Integrated Microbial Genomes, (https://img.jgi.doe.gov) by using pfam12288 as search term. There were a total of 165 CsoS2 proteins available as of September 2014. The computation of molecular weight and the theoretical pI (isoelectric point) was carried out using online Compute pI/Mw tool (http://web.expasy.org/compute_pi/). Multiple sequence alignment was performed using T-Coffee at EMBL-EBI server [24,61]. Based on alignment, each CsoS2 sequence was roughly divided into fragments each including a single N- or M-region repeat, or the C-terminal region. All the N-region fragments were pooled together and used as input for the motif discovery tool MEME (http://meme.nbcr.net/meme) [62]. Similarly, all the M-region fragments were pooled and submitted to MEME server. Two motifs with different lengths were identified in this case, each corresponding to the long and short form. The long form is 50 amino acids long and was identified with an E-score of 4.3 × e^−11,260^ in 474 out of 605 input sequences, while the short form is 40 amino acids long with an E-score of 1.8 × e^−10,176^, found in 554 out of 605 input sequences. Pairwise alignments of full-length or C-terminal region between 7 representative CsoS2s were performed by using online EMBOSS Needle algorithm (http://www.ebi.ac.uk/Tools/psa/emboss_needle/) [61,63].

### 4.2. Prediction of Secondary Structure and Protein Folding

Secondary structure was predicted using Jpred3 (http://www.compbio.dundee.ac.uk/www-jpred/) [64]. Folding potential was evaluated by the feed-forward neural networks based prediction algorithm, PONDR (http://pondr.com) [65,66] as well as FoldIndex (http://bip.weizmann.ac.il/fldbin/findex) [67]. *Ab initio* protein structure prediction for each M-repeat with flanking sequences was performed at the QUARK server (http://zhanglab.ccmb.med.umich.edu/QUARK/) [68,69].

### 4.3. Construction of Hnea csoS2::Km^R^ Mutant

The *Hnea csoS2* gene was interrupted by inserting the *Km^R^* gene cassette into the EcoRV site of *csoS2* (cut at nucleotide #507 in the ORF of *csoS2*) (Figure 3a). Briefly, the *Km^R^* cassette was cut from pUC4K with EcoRI and the ends were polished with Klenow enzyme to create a blunt-ended fragment. This fragment was inserted into pTn1-2SE1.3 at the EcoRV site via blunt-end ligation. The resulting plasmid was confirmed with restriction digest and then named pTn1-2SE1.3Km4K. Wildtype mid-exponential phase *Hnea* cells were electroporated with pTn1-2SE1.3Km4K plasmid as previously described [11], and transformants were selected in the presence of 10 µg/mL kanamycin and in 5% CO_2_ enriched air. The genomic DNA of the positive clone was confirmed by sequencing.

### 4.4. Construction of HnSPAS2 Mutant

A pair of primers was designed to amplify the SPA tag and a *Km^R^* gene cassette by PCR, using the *pJL148* plasmid [70] as the template. To create an in-frame fusion between the PCR product and the 3' end of the *Hnea* *csoS2* coding sequence by homologous recombination, the forward primer contained 45 bp of nucleotide sequence corresponding to the region of the *csoS2* gene immediately upstream from the stop codon, followed by the first 17 bp of the SPA tag sequence. The reverse primer contained 45 bp of nucleotide sequence immediately downstream of the *csoS2* stop codon, followed by the last 20 bp of the *Km^R^* gene. The *E. coli* DY330 strain was co-transformed with the resulting PCR product and with *pTncsoS2::csoS3* [71,72]. This strain carries the λ phage Red recombinase gene that facilitates precise homologous recombination between very short 45 bp homologous regions and was used to produce the SPA tagged version of *csoS2*, followed by a *Km^R^* gene cassette in the *pTncsoS2::csoS3SPA* plasmid. In this plasmid, the SPA tag and *Km^R^* gene cassette are flanked by the long (approximately 2000 bp) homologous regions that facilitated the subsequent gene replacement in Hnea by homologous recombination. Wildtype mid-exponential phase *Hnea* cells were electroporated with the *pTncsoS2::csoS3SPA* plasmid and transformants were selected in the presence of 50 µg/mL kanamycin and in 5% CO_2_ enriched air. The genomic DNA of two positive clones was confirmed by sequencing, and one of these clones was used to establish a working culture. The resulting mutant was named *HnSPAS2*.

### 4.5. Purification of HnSPAS2 carboxysomes

Wildtype and SPAS2 mutant *Hnea* cells were grown in a bioreactor at 0.08 h^−1^ dilution rate at pH 6.4 and 30 °C in an ambient CO_2_ environment. Eight liters of cells were concentrated to 500 mL with a Pellicon™-2 cassette filter (Millipore, Billerica, MA, USA) and pelleted by centrifugation at 12,000 g for 10 min. The cell pellet was resuspended in 20 mL of TEMB buffer (10mM Tris-HCl, pH 8.0, 1 mM EDTA, 15 mM MgCl_2_, 20 mM NaHCO_3_) supplemented with 1 mg/mL lysozyme and 5 mg/mL MgSO_4_. The suspension was mixed with an equal volume of B-PER II detergent (Pierce, Waltham, MA, USA) and sonicated for 10 to 15 s until the suspension became less viscous. The resulting cell lysate was mixed with 200 Kunitz units of DNase I (Sigma, St. Louis, MO, USA) and gently agitated for 30 min at room temperature. Cell debris was removed by centrifugation at 10,000 g for 10 min at 4 °C. The resulting supernatant was spun at 48,000 g for 30 min at 4 °C to obtain a carboxysome-enriched pellet. This pellet was resuspended in 3 mL of TEMB buffer and loaded onto a linear 10%–60% sucrose gradient prepared in TEMB buffer. Centrifugation at 100,000 g for 30 min at 4 °C in a Beckman JS-24.38 rotor yielded a narrow white band of carboxysomes in the middle of the gradient. This band was collected and the carboxysomes were pelleted by centrifugation at 126,000 g for 2 h at 4 °C. The carboxysome pellet was resuspended in 0.5 mL of TEMB buffer and stored at 4 °C.

### 4.6. Yeast Two Hybrid Assay

Recombinant protein expression in yeast was accomplished using bait or prey expression vectors, *pGBKT7* and *pGADT7* respectively, provided as part of the Matchmaker GAL4 Two-Hybrid System 3 (Clontech, Palo Alto, CA). All carboxysome genes were individually amplified by PCR using *pTnl* template DNA [73]. Designed primers for the yeast two-hybrid system (Table S6) were used to add endonuclease restriction sites Ndel and BamHI that flank the 5' and 3' ends of the PCR products, which allowed for the subcloning into either bait or prey vector DNA. *Saccharomyces cerevisiae* AH 109 yeast cells were prepared for transformation by first streaking solid YPDA with frozen AH109 yeast stock and incubating at 30 °C until 2–4 mm colonies appeared. A single 3 mm colony was selected and transferred to 3 mL of liquid YPDA medium in a sterile 15 mL centrifuge tube. The culture was incubated at 30 °C while shaking for 8 h at 250 rpm. 5 mL of the culture was transferred to a 250 mL Erlenmeyer flask containing 50 mL of YPDA and incubated overnight for 16–20 h at 30 °C and 250 rpm. Culture density of OD_600_ between 0.15 and 0.3 was verified before the cells were recovered by centrifugation at 700 g for 5 min, 25 °C. The cell pellet was recovered in 100 mL of YPDA and incubated at 30 °C for 3–5 h. The cells transformed with *pGADT7* constructs were maintained on synthetic complete (SC) medium lacking leucine and supplemented with 0.01% adenine. Yeast cells transformed with *pGBKT7* constructs were maintained on SC medium lacking tryptophan and supplemented with 0.01% adenine. Co-transformed yeast cells that contained both *pGADT7* and *pGBKT7* were maintained on SC medium lacking tryptophan and leucine and supplemented with 0.01% adenine. To observe protein-protein interactions *in vivo*, liquid cultures of single or co-transformed AH109 cells were grown to OD_590_ of 0.6 to 0.8, then plated onto selective solid SC medium containing 80 ng/mL 5-Bromo-4-chloro-3-indolyl-α-d-galactopyranoside (X-α-Gal). Single transformed colonies plated on analogous selection media were screened for transcriptional activation of the reporter gene in the absence of the complementing co-transformed plasmid. *In vivo* bait-prey interactions were analyzed by observing blue colony formation due to transcription/translation of the MEL1 reporter gene. To determine protein-protein interactions using nutrient selection, AH1 09 yeast cells were grown on triple selective solid SC medium lacking tryptophan, leucine and histidine and supplemented with 0.01% adenine and 10 mM 3-amino-1,2,4-triazole (3-AT). Bait-prey interactions in yeast maintained on triple selective media were visually analyzed for colony formation due to transcription/translation of the His3 protein, which allowed yeast cells to grow in the absence of histidine.

### 4.7. Characterization of Biophysical Properties

Prior to SAXS data collection, purified recombinant proteins MED4 CsoS2, MIT9313 CsoS2-NM, MIT9313 RuBisCO and CsoS1 were subjected to size exclusion chromatography using a Superdex 200 column (GE Healthcare, Little Chalfont, UK) buffered with 50 mM HEPES, 300 mM NaCl (pH 7.4) with the exception of RuBisCO, where RuBisCO storage buffer (see above) was used. All protein samples were concentrated using Amicon Ultra-0.5 mL 3K device (Millipore, Billerica, MA, USA) to protein concentrations of 4.5, 10.8, 6.9 and 1.8 mg/mL, respectively (measured by BCA assay, Pierce, Waltham, MA, USA). Then 1:2 and 1:5 dilutions of each protein was used in SAXS experiment as Medium (M) and Low (L) concentration, together with the un-diluted sample (High concentration). SAXS experiments were performed at the SIBYLS beamline 12.3.1 at the Advanced Light Source at Lawrence Berkeley National Laboratory. For each sample, scattering intensities were measured at the three protein concentrations (H, M and L). Data were collected for each protein concentration at exposure times of 0.5, 1.0 and 3.0 s. The scattering curves obtained from the protein samples were corrected for background scattering using intensity data collected from the reference buffer.

The SAXS data were analyzed using the ATSAS program suite (version 2.4, EMBL Hamburg, Hamburg, Germany) [74]. Data collected for each protein sample was consolidated in PRIMUS [75] by scaling and merging the background-corrected low-*q* region data from the 0.5 or 1.0 s exposure with the high-*q* region data from the 3.0 s exposure. GNOM was used to calculate pair distribution functions (*P<r>* function) [76].

Prior to CD measurements, MED4 CsoS2 was exchanged into 10 mM potassium phosphate pH 7.4, 50 mM (NH_4_)_2_SO_4_ by performing multiple buffer exchanges in a centrifugal spin concentrator (Amicon Ultra 10K, Millipore, Billerica, MA, USA). Far UV-CD spectra were acquired at 4 °C in a 0.1 cm path length cuvette using a Jasco J-815 spectropolarimeter. Individual scans (260 to 190 nm) were collected with a 2 s time constant, 20 nm/min scan speed, and 1 nm bandwidth. Eight scans were averaged to produce the final spectrum. Protein concentration (170 µg/mL) was determined using the molar extinction coefficient of CsoS2 at 280 nm and the mean residue ellipticity, [θ] (in deg cm^2^ dmol^−1^), was calculated from the raw CD signal (millidegrees) using the following equation: [θ] = (CD signal [millidegrees] × Mean Residue Weight [molecular weight/number of backbone amides])/(path length [mm] × concentration [mg/mL]).

Size exclusion chromatography of CsoS2 proteins was performed using Bio-Rad BioLogic DuoFlow FPLC system and a Superdex 200 column (GE Healthcare, Little Chalfont, UK) buffered with 50 mM HEPES, 300 mM NaCl (pH 7.4). The turbidity upon mixing CsoS2 and CsoS1 was measured by OD at 600 nm, and ΔOD_600_ calculated as:

$$\text{∆}OD_{600\text{nm}}=OD_{600\text{nm after mixing}}\;-\frac{OD_{600\text{nm CsoS}2}\;\times \;{\text{volume}}_{\text{CsoS}2}\;+\;OD_{600\text{nm CsoS}1}\;\times \;{\text{volume}}_{\text{CsoS}1}}{{\text{volume}}_{\text{CsoS}2}\;+\;{\text{volume}}_{\text{CsoS}1}}$$

### 4.8. Peptide Array

An 8-mer tiling peptide library with one amino acid walking and at least four redundancies to span the entire sequence of MIT9313 CsoS2 was synthesized by LC Sciences (Houston, TX, USA) using the PepArray technology [77]. The peptides were synthesized *in situ* on microfluidic chips; three identical chips were designated for each carboxysome protein in binding assay: RuBisCO holoenzyme, CsoS1 and CsoS1D. Binding assays were performed by LC Sciences. Briefly, each chip was pre-blocked overnight at 4 °C and then incubated with 10 µg/mL of each tested protein in 1X TBS pH 7.0 at 4 °C for 4 h and washed in same buffer for 20 min. Then the chip was incubated with 100 ng/mL of antibody anti-His w. Alexa 647 conjugation (for CsoS1, CsoS1D) or 100 ng/mL rabbit anti-RbcL for RuBisCO plus 10 ng/mL goat anti-rabbit IgG with Alexa 647 conjugation for another 4 h at 4 °C. Scanning of the chip was performed using Microarray scanner PMT700 at 635 nm. Raw images after binding assay are shown in Figure S5. False coloring was applied to facilitate imaging of signal intensities with blue for no binding to red for maximal binding. To be counted as detectable a spot must meet two conditions: (1) signal intensity after background subtraction is higher than 3 times of background standard deviation; (2) standard deviation divided by signal intensity is less than 0.5. A peptide is counted as a positive hit only if more than 50% of replicate spots are detectable spots in the previous step.

## Supplementary Files

Supplementary File 1
