# Supplementary material for: Advances in Understanding Carboxysome Assembly in Prochlorococcus and Synechococcus Implicate CsoS2 as a Critical Component
Source: Life (Basel). 2015 Mar 27;5(2):1141–71. doi: 10.3390/life5021141 (PMC4499774; doi:10.3390/life5021141)
Supplement: Supplementary File 1 [file life-05-01141-s001.zip › Supplementary.pdf]

Supplemental Information

S1. Supplemental Materials and Methods

S1.1. Cloning, Strains and Culturing Conditions

Expression constructs used in this study for recombinant protein expression are listed in Table S2. Cloning was done via standard digestion and ligation procedures using *Hnea*, MED4 or MIT9313 genomic DNA as a template and primers listed in Table S5. Codon optimized MED4 *csoS1*, *csoS1D* and *csoS2* genes were synthesized at GenScript (Piscataway, NJ) and then sub-cloned into pCDFDuet-1 vector (Novagen, Madison, WI, USA). The sequences of the protein coding regions of each expression construct were confirmed by DNA sequencing the (University of California at Berkeley DNA sequencing facility). If not specified, all protein expressions were performed with *Escherichia coli* BL21(DE3) cells (Invitrogen, Carlsbad, CA, USA). *E. coli* transformed with expression constructs were grown in LB (EMD Millipore, Billerica, MA) media at 37 °C with appropriate antibiotics (100 µg/mL Ampicillin or 50 µg/mL Spectinomycin or 35 µg/mL Chloramphenicol). IPTG (Gold Biotechnology, St. Louis, MO, USA) was added to a final concentration of 0.4 mM when cultures had grown to OD600 ≈ 0.6–0.7 to induce recombinant protein expression. Cultures continued to be grown at 25 °C for 3 h before harvesting. Following 15 min centrifugation at 5000 g, cell pellets were weighed and stored in −20 °C until purification. Heterogeneous expression of recombinant MIT9313 RuBisCO was done as previously described [1]. Wildtype or mutant *Hnea* strains were grown as previously described [2].

S1.2. Recombinant Protein Purification

Cell pellet with expression of His-tagged *Hnea* CsoS2 was resuspended in lysis buffer (50 mM Tris-HCl, pH 8.0, 5 mM β-ME, 1 mM PMSF/PTSF) and the cells were lysed by 20 30-s cycles of sonication in an ice bath. Cell debris was removed by centrifugation at 20,000 g for 30 min. The supernatant was transferred to a new tube and mixed with 3 mL of Ni-NTA beads equilibrated with lysis buffer. The slurry was incubated for at least 3 h. The beads were washed with 20 bed volumes of low salt washing buffer (20 mM Tris-HCl, pH 8.0, 100 mM NaCl, 5 mM β-ME, 10% glycerol, 20 mM imidazole, 0.1 mM PMSF/PTSF), 10 bed volumes of high salt washing buffer (20 mM Tris-HCl, pH 8.0, 1 M NaCl, 5 mM β-ME, 10% glycerol, 0.1 mM PMSF/PTSF) and 5 bed volumes of low salt washing buffer sequentially. The His-tagged CsoS2 protein bound to the beads was eluted with 20 mL of elution buffer (20 mM Tris-HCl, pH 8.0, 100 mM NaCl, 5 mM β-ME, 10% glycerol, 250 mM imidazole, 0.1 mM PMSF/PTSF). Ten 2-mL elution fractions were collected and analyzed by SDS-PAGE. All fractions containing recombinant CsoS2 protein were pooled and dialyzed against two changes of 4 liters of dialysis buffer (10 mM Tris-HCl, pH 8.0, 20 mM NaCl and 10 mM β-ME).

Cell pellet with expression of *Hnea* CsoS2 with C-terminal self-splicing intein-CBD tag was resuspended in 50 mL of lysis buffer (20 mM Na_2HP0_4, pH 8.0, 500 mM NaCl, 1 mM EDTA and 1 mM PMSF/PTSF) and disrupted by passage through a French press cell at 20,000 psi. The lysate was clarified by centrifugation at 20,000 g for 30 min. The resulting supernatant was mixed with 20 mL of chitin beads equilibrated in lysis buffer and incubated overnight. The slurry was washed with 10 bed volumes of low salt wash buffer (20 mM Na_2HP0_4, pH 8.0, 500 mM NaCl, 1 mM EDTA and 0.1 mM

PMSF/PTSF), 20 bed volumes of high salt wash buffer (20 mM Na_2HP0_4, pH 8.0, 2 M NaCl, 1 mM EDTA and 0.1 mM PMSF/PTSF), 10 bed volumes of low salt wash buffer and 3 bed volumes of cleavage buffer (20 mM Na_2HP0_4, pH 8.0, 500 mM NaCl, 1 mM EDTA, 50 mM DTT and 0.1 mM PMSF/PTSF). The fusion protein was allowed to self-cleave on the column over a 24 h period at 4°C and eluted with 70 mL of low salt wash buffer. Ten 7 mL fractions were analyzed by SDS-PAGE. All fractions containing recombinant CsoS2 were pooled and placed into a 3,500 molecular weight cut off dialysis bag. The dialysis bag was covered with polyethyleneglycol (PEG) 8,000 powder to absorb any buffer leaving the dialysis bag at room temperature, and absorption was allowed to proceed until the final volume of the sample inside the bag had decreased to 1 ml. Residual PEG was rinsed off with deionized water prior to dialyzing the concentrated CsoS2 protein against 4 liters of dialysis buffer (10 mM Tris-HCl, pH 8.0, 20 mM NaCl and 10 mM β-ME) overnight at 4 °C.

GST-tagged full-length MED4 CsoS2 or fragments of MIT9313 CsoS2 were purified using a cation exchange column followed by affinity column. Briefly, the cell pellet was resuspended in 50 mM HEPES pH 7.4 (20 mL per 1 liter culture) with protease inhibitor, cOmplete MINI (Roche Life Science, Indianapolis, IN, USA). Cells were lysed with a French press; supernatant obtained after centrifugation at 40,000 g for 30 min at 4 °C was applied onto 10 ml Sulphopropyl Sepharose Fast Flow resin (GE Healthcare, Pittsburgh, PA, USA). After washing with two column volumes of 50 mM HEPES pH 7.4, a linear gradient was used with 0 to 1 M NaCl. GST-tagged CsoS2 proteins were collected at 325 mM NaCl concentration and applied onto Glutathione Sepharose resin (GE Healthcare, Little Chalfont, UK). Affinity purification was performed according to manufacturer's protocol. GST-tagged full-length MED4 CsoS2 was eluted using 10 mM reduced glutathione. In all other cases, on-column cleavage of GST-tag was performed using PreScission Protease (PSP) (GE Healthcare, Little Chalfont, UK) following manufacturer's protocol.

StrepII-tagged MIT9313 CsoSCA purification was similar to the above procedure for GST-tagged CsoS2 purification, except that DEAE Sepharose column was followed by Strep Tactin column (Novagen) according to manufacturer's protocol. Recombinant protein was eluted with the presence of 2.5 mM desthiobiotin.

MIT9313 CsoS1 protein was purified as previously described using HisTrap HP column (GE Healthcare, Little Chalfont, UK) [3]. MIT9313 CsoS1D, CsoS1E and $CsoSCA_{1\text{-}145}$ purifications were performed using the same procedure.

Recombinant MIT9313 RuBisCO holoenzyme was purified following Scott et al. paper [1], with some modifications inspired by a virus capsid protein purification protocol [4]. Briefly, after 20%–55% $(NH_4)_2SO_4$ precipitation step, precipitated protein was re-dissolved with RuBisCO storage buffer (50 mM Bicine, pH 7.5, 30 mM $MgCl_2$, 66 mM $NaHCO_3$, 5 mM dithiothreitol, 20% Glycerol) and applied to a 1-mL Resource Q (GE Healthcare, Little Chalfont, UK) column with a gradient of 0 to 1 M NaCl. Fractions containing RuBisCO were pooled and run on an S200 size exclusion column in RuBisCO storage buffer, and peak fractions were pooled. This pool was brought to 2 mM ATP, 5 mM $MgCl_2$ and 5mM DTT and stirred for 1 hour at 4 °C. Then solid urea was slowly added to a final concentration of 1 M and the sample was stirred overnight at 4 °C. The sample was then dialyzed against two changes of buffer (50 mM HEPES pH 7.5, 20 mM $MgCl_2$, 1 mM ATP, 5 mM DTT and 10% glycerol). This sample was again applied to Resource Q column. Presence of RuBisCO and

absence of GroES/EL were confirmed by Western Blots using antibodies α-RbcL (form I & II) (Agrisera, Sweden), α-GroEL and α-GroES (Abcam, Cambridge, MA, USA).

S1.3. Protein SDS-PAGE and Western Blots

Protein samples were separated on pre-cast 4%–20% or 10%–20% sodium dodecyl sulfate polyacryamide gradient gels (BioRad, Hercules, CA, USA) to analyze their composition. Polypeptide bands were visualized by staining with Gel Code Blue (Pierce, Waltham, MA, USA). For immunoblotting, the proteins were transferred onto a 0.45 µm pore size nitrocellulose membrane in a Mini Trans-Blot electrophoretic transfer cell (BioRad, Hercules, CA, USA). The blot was blocked with immunoblot blocking buffer (5% nonfat milk in PBS or TBS with 0.1% Triton X-100) for 30 min. The appropriate primary antibody generated in rabbits was incubated with the blot for 1 h to probe the presence of the target antigen. After rinsing with PBS buffer, immunoblot blocking buffer and PBS or TBS buffer for 15 minutes each, the blot was incubated with goat anti-rabbit IgG antibody conjugated with alkaline phosphatase (AP) or horseradish peroxidase (HRP) for 1 h. The blot was developed with one step NBT-BCIP solution for chromatic detection of AP activity or SuperSignal® West Pico Chemiluminescent Substrate for HRP detection (Pierce, Waltham, MA, USA). Images of stained gels and immunoblots were captured and documented using a VersaDoc imaging system model 4000MP or ChemiDoc imaging system (BioRad, Hercules, CA, USA). Densitometry analysis was performed using the Quantity One program or ImageLab program (BioRad, Hercules, CA, USA).

S1.4. Transmission Electron Microscopy

Thin sections of *Hnea csoS2::Km*^{R} mutant strain were prepared and imaged as previously described [5]. A suspension of purified carboxysomes was placed onto formvar/carbon coated copper grids and allowed to stand for 3 min. After drying the samples in air for 10 s, the samples were negatively stained with 1% (w/v) ammonium molybdate in 10 mM Tris-HCl, pH 8.0 for 40 s. The grids were air-dried for 10 min before being observed under a Zeiss EM-109 transmission electron microscope (Carl Zeiss AG, Jena, Germany. Images of carboxysomes were taken by exposing Kodak EM film 4489 for 2 s.

S1.5. MALDI-ToF Analysis of Hnea CsoS2A and CsoS2B

Polypeptides of wildtype *Hnea* carboxysomes were separated by SDS-PAGE. Gels were stained with 0.05% aqueous Coomassie Brilliant Blue R250 solution for 1 h, and destained with deionized water several times until the target band was visible. The protein band of interests was excised and destained twice for 45 min each with 200 µL of 100 mM NH_4HCO_3/50% acetonitrile (ACN) at 37 °C to remove trace amounts of remaining dye. The gel slice was dehydrated in 100 µL of 100% ACN for 5 min at room temperature and the ACN was removed by vacuum centrifugation in a SpeedVac for 10–15 min. The dry gel slice was rehydrated in 10–20 µL of a 20 µg/mL trypsin solution (Promega, Madison, WI, USA) in 40 mM NH_4HCO_3/10% ACN at room temperature for 1 h. A volume of digestion buffer (40 mM NH4HCO3/10% ACN) sufficient to cover the gel slice was added and the sample was incubated at 37 °C overnight. The gel slice was removed out and incubated in deionized

water for 10 min with frequent vortex mixing. The liquid was transferred to a new tube, and the gel slice was extracted twice with 50 µL of 50% ACN/5% trifluoroacetic acid (TFA) at room temperature for 60 min each time. All extracts were pooled and dried in a SpeedVac for 2–4 h at room temperature. The resulting dry white powder was reconstituted with 10 µL of 0.1% TFA and the peptide mixture was purified and concentrated with ZipTip C^{18} pipette tips (Millipore, Billerica, MA, USA) prior to mass spectrometric analysis according to the manufacturer's instruction. The Mascot program was used to analyze the mass spectrometry data. All mass lists were searched against the NCBI non-redundant protein database.er

S1.6. In vitro Pull-Down Assay of Recombinant carboxysome Proteins

Escherichia coli strain BL21-CodonPlus (DE3)-RIL (Stratagene) was co-transformed with pGEX-MED4*csoS2* and pCDFDuet-MED4*csoS1D*/*csoS1* or pCDFDuet-MED4*csoS1*, and single colonies were picked from LB-agar plate with appropriate antibiotics. Protein expression was performed as described above. Cell pellets each from 150 ml culture were collected and kept frozen at −20 °C until usage. 800 µL of B-PER II (2X) reagent (Pierce Biotechnology, Rockford, IL, USA) was added to each sample to resuspend cell pellet, followed by 15 min shaking at room temperature. Then 800 µl of lysis buffer (500 µL of GST-Pulldown Buffer; 75 µL of 10X protease Inhibitor Stock; 75 µL of 10 mg/mL lysozyme; and 150 µL of 2 U/µL DNase I) was added to each sample and followed by another 15 min shaking at room temperature. The GST-Pulldown Buffer is composed as follows: 125 mM Tris-HCl, pH 8.0, 150 mM NaCl, 10% Glycerol and 1% NP-40. The cell lysate was spun at maximum speed for 15 min at 4 °C in a bench-top Eppendorf centrifuge. The supernatant was saved and applied to 50 µL of pre-equilibrated Glutathione-Sepharose magnetic beads (Pierce, Waltham, MA, USA). Incubation was carried out at 4 °C with rotation overnight. The rest of the experiment was performed as previously described [6].

In vitro pull-down from cells expressing MED4 CsoS2-his_6, CsoS1 and CsoS1D from pMED04 using Ni-NTA magnetic agarose beads (Qiagen, Valencia, CA, USA) was also performed in a similar manner except different buffers were used. Lysis/wash buffer is composed as following: 20 mM MOPS, pH 8.0, 300 mM KCl, 20 mM imidazole and 0.05% (v/v) Tween 20; the elution buffer has 500 mM imidazole instead of 20 mM.

S1.7. Enrichment of Complex Formed by Recombinant carboxysome Proteins

Expression of pMED04 was performed in *E. coli* BL21(DE3) cells as described earlier. Cell pellet was weighed, and the same value in ml of buffer (40 mM KH_2PO_4, 260 mM K_2HPO_4 and 500 mM Sucrose) was added to resuspend the cell. Then 200 µL of DNaseI at 2 U/µL, 200 µL of 10 mg/mL RNase A and 100 µL of 10 mg/mL lysozyme was also added per 5 g cell pellet weight. At this point, B-PER Phosphate reagent (Pierce, Waltham, MA, USA) was added to double the volume. Mixture was incubated on a shaker for 30 min at room temperature. Cell debris was removed with an initial centrifugation at 9000 rpm in rotor SS-34 for 15 min at 4 °C. 1.5% (w/v) of Octyl β-D-Glucopyranoside (OBDG) was added to the supernatant and incubated on a shaker for 30 min at 4 °C. A second centrifugation step was performed at 20,000 rpm in rotor SS-34 for 30 min at 4 °C.

Supernatant was discarded, and the glassy pellet was briefly washed with phosphate buffer (40 mM KH_2PO_4, 260 mM K_2HPO_4), then resuspended in 1 mL; of phosphate buffer.

S1.8. Protein agarose gel

Protein agarose gel (1% w/v) was prepared and run in BEMB buffer (20 mM Bicine, 1 mM EDTA, 10 mM $MgCl_2$ and 20 mM $NaHCO_3$) pH 7.2. *Hnea* rCsoS2 (0.6 mg/ml), *Hnea* RuBisCO (native, released from *Hnea* carboxysomes; 1.1 mg/mL) and *Hnea* rCsoS1A (0.7 mg/mL) were individually dialyzed against BEMB pH 7.2 before the experiment.

S1.9. Estimations of the local protein concentration in α-carboxysomes

A typical α-carboxysome (ex. a *Hnea* carboxysome) has a diameter of approximately 100 nm, and is considered a perfect icosahedron, which gives the sphere radius ***r*** as 50 nm. And based on (where ***a*** is the edge length and ***V*** is the volume):

$$\boldsymbol{r} = \frac{\boldsymbol{a} * \sqrt[2]{10 + 2\sqrt[2]{5}}}{4}$$

$$\boldsymbol{V} = \frac{\boldsymbol{a}^3 * 5(3 + \sqrt[2]{5})}{12}$$

A typical α-carboxysome has volume of $3.17 \times 10^{\mathbf{-16}}$ cm^3, which is equal to $3.17 \times 10^{\mathbf{-16}}$ mL.

CsoS2 has a calculated MW of 92 kDa, where 1 Da is equal to $1.66 \times 10^{\mathbf{-21}}$ mg; and there are approximately 330 copies of CsoS2 per carboxysome [7]. So each carboxysome has approximately $5.04 \times 10^{\mathbf{-14}}$ mg of CsoS2, which means the local concentration of CsoS2 is approximately 159 mg/ml. Similarly, the local concentration of RuBisCO (270 L_8S_8, MW_{cal} of holoenzyme is 520.8 kDa) inside of carboxysomes can be calculated as approximately 735 mg/mL.

2. Supplemental Figures

a

```
  1 MPSQSGMNPA DLSGLSGKEL ARARRAALSK QGKAAVSNKT ASVNRSTKQA
 51 ASSINTNQVR SSVNEVPTDY QMADQLCSTI DHADFGTESN RVRDLCRQRR
101 EALSTIGKKA AKTTGKPSGR VRPQQSVVHN DAMIENAGDT NQSSSTSLNN
151 ELSEICSIAD DMPERFGSQA KTVRDICRAR RQALSERGTR AVPPKPQSQG
201 GPGRNGYQID GYLDTALHGR DAAKRHREML CQYGRGTAPS CKPTGRVKNS
251 VQSGNAAPKK VETGHTLSGG SVTGTQVDRK SHVTGNEPGT CRAVTGTEYV
301 GTEQFTSFCN TSPKPNATKV NVTTTARGRP VSGTEVSRTE KVTGNESGVC
351 RNVTGTEYMS NEAHFSLCGT AAKPSQADKV MFGATARTHQ VVSGSDEFRP
401 SSVTGNESGA KRTITGSQYA DEGLARLTIN GAPAKVARTH TFAGSDVTGT
451 EIGRSTRVTG DESGSCRSIS GTEYLSNEQF QSFCDTKPQR SPFKVGQDRT
501 NKGQSVTGNL VDRSELVTGN EPGSCSRVTG SQYGQSKICG GGVGKVRSMR
551 TLRGTSVSGQ QLDHAPKMSG DERGGCMPVT GNEYYGREHF EPFCTSTPEP
601 EAQSTEQSLT CEGQIISGTS VDASDLVTGN EIGEQQLISG DAYVGAQQTG
651 CLPTSPRFNQ TGNVQSMGFK NTNQPEQNFA PGEVMPTDFS IQTPARSAQN
701 RITGNDIAPS GRITGPGMLA TGLITGTPEF RHAARELVGS PQPMAMAMAN
751 RNKAAQAPVV QPEVVATQEK PELVCAPRSD QMDRVSGEGK ERCHITGDDW
801 SVNKHITGTA GQWASGRNPS MRGNARVVET SAFANRNVPK PEKPGSKITG
851 SSGNDTQGSL ITYSGGARG
```

b

```
  1 MPSQSGMNPA DLSGLSGKEL ARARRAALSK QGKAAVSNKT ASVNRSTKQA
 51 ASSINTNQVR SSVNEVPTDY QMADQLCSTI DHADFGTESN RVRDLCRQRR
101 EALSTIGKKA AKTTGKPSGR VRPQQSVVHN DAMIENAGDT NQSSSTSLNN
151 ELSEICSIAD DMPERFGSQA KTVRDICRAR RQALSERGTR AVPPKPQSQG
201 GPGRNGYQID GYLDTALHGR DAAKRHREML CQYGRGTAPS CKPTGRVKNS
251 VQSGNAAPKK VETGHTLSGG SVTGTQVDRK SHVTGNEPGT CRAVTGTEYV
301 GTEQFTSFCN TSPKPNATKV NVTTTARGRP VSGTEVSRTE KVTGNESGVC
351 RNVTGTEYMS NEAHFSLCGT AAKPSQADKV MFGATARTHQ VVSGSDEFRP
401 SSVTGNESGA KRTITGSQYA DEGLARLTIN GAPAKVARTH TFAGSDVTGT
451 EIGRSTRVTG DESGSCRSIS GTEYLSNEQF QSFCDTKPQR SPFKVGQDRT
501 NKGQSVTGNL VDRSELVTGN EPGSCSRVTG SQYGQSKICG GGVGKVRSMR
551 TLRGTSVSGQ QLDHAPKMSG DERGGCMPVT GNEYYGREHF EPFCTSTPEP
601 EAQSTEQSLT CEGQIISGTS VDASDLVTGN EIGEQQLISG DAYVGAQQTG
651 CLPTSPRFNQ TGNVQSMGFK NTNQPEQNFA PGEVMPTDFS IQTPARSAQN
701 RITGNDIAPS GRITGPGMLA TGLITGTPEF RHAARELVGS PQPMAMAMAN
751 RNKAAQAPVV QPEVVATQEK PELVCAPRSD QMDRVSGEGK ERCHITGDDW
801 SVNKHITGTA GQWASGRNPS MRGNARVVET SAFANRNVPK PEKPGSKITG
851 SSGNDTQGSL ITYSGGARG
```

Figure S1. Mapping of *Hnea* CsoS2A and CsoS2B derived peptides by MALDI-TOF mass spectrometry. The polypeptides of purified *Hnea* carboxysomes were separated by the SDS-PAGE and the CsoS2A (**a**) and CsoS2B (**b**) bands were subjected to in-gel digestion with trypsin. Peptides marked in red are identified in the digested protein mixtures by MALDI-ToF mass spectrometry. CsoS2B protein was traced to the arginine residue next to the last amino acid of the deduced sequence of *Hnea csoS2* gene, which indicated that it is a full length CsoS2 protein.

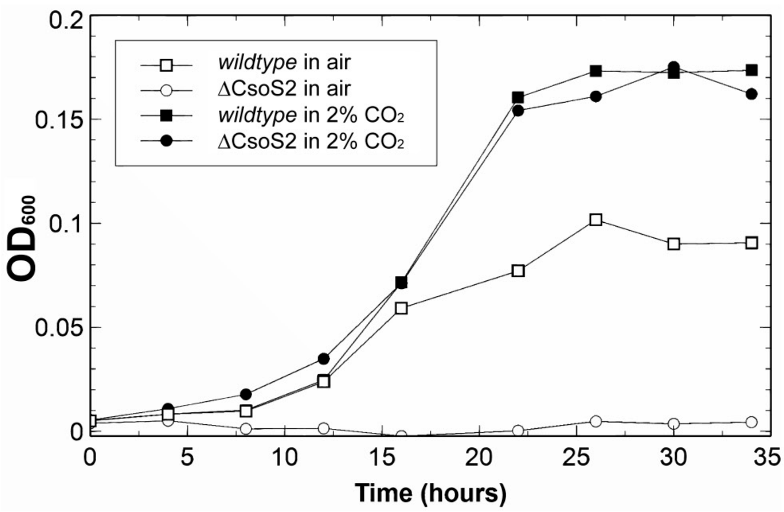

Figure S2. Growth of *Hnea* ΔCsoS2 mutant. *Hnea* ΔCsoS2 mutant cells grow similarly to wildtype cells in 2% CO_2 but does not grow in air.

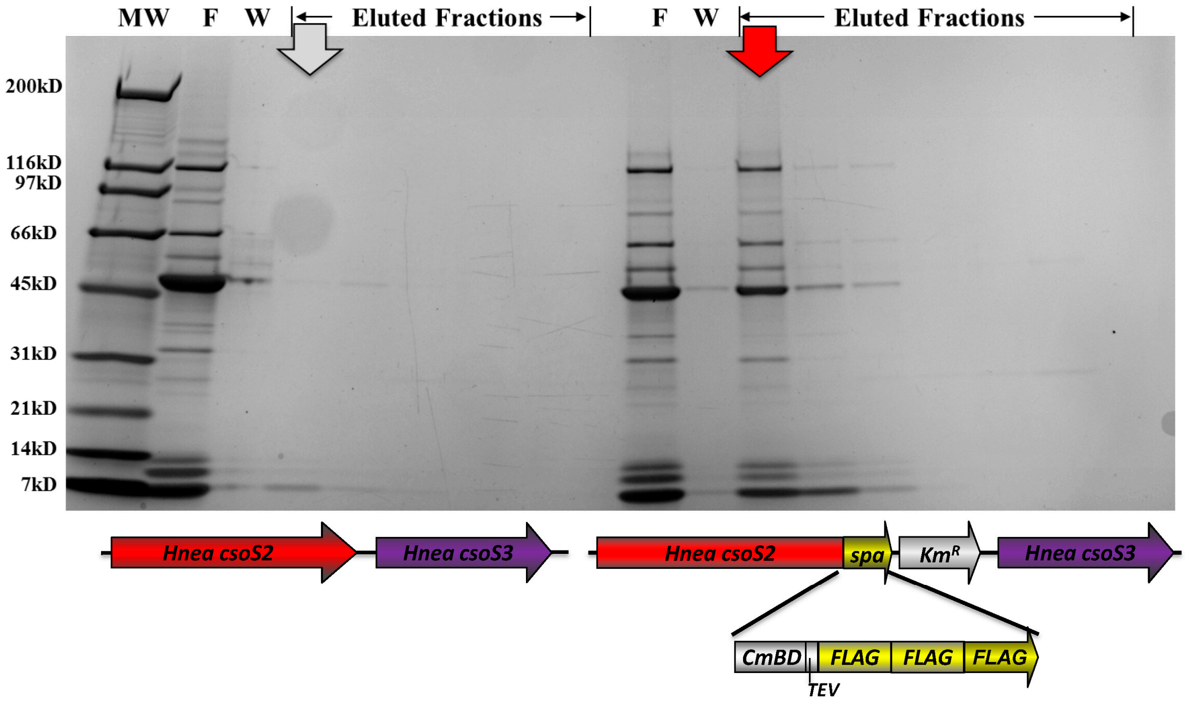

Figure S3. Trapping of SPA-tagged *Hnea* carboxysomes on an α-FLAG affinity column. Isolated wildtype *Hnea* and *HnSPAS2* mutant carboxysomes are mixed with α-FLAG affinity beads. The trapped carboxysomes were eluted with 100 mM glycine buffer, pH 2.5. MW: protein molecular weight standards; FT: flow-through fraction; Wash: last wash fraction; E: elution fraction. Purified *HnSPAS2* carboxysomes bound to the affinity column, while wildtype carboxysomes had no interaction with the resin, which suggests that the C-terminus of CsoS2 protein may be exposed to the outer surface of the carboxysome shell. There were extremely low amounts of polypeptide with an apparent molecular weight of approximately 7 kDa in the first elution fraction for wildtype carboxysomes, which may represent the nonspecific binding of broken carboxysome shell component with the affinity column.

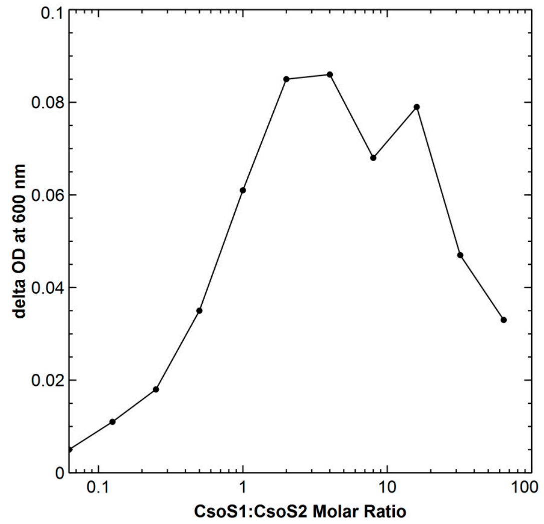

Figure S4. Increases in opacity when MED4 CsoS2 is combined with MED4 CsoS1. Purified MED4 CsoS2 (~6 mg/mL) is mixed with purified MED4 CsoS1 protein at molar ratios from 16:1 to 1:64. Increase in opacity is observed immediately.

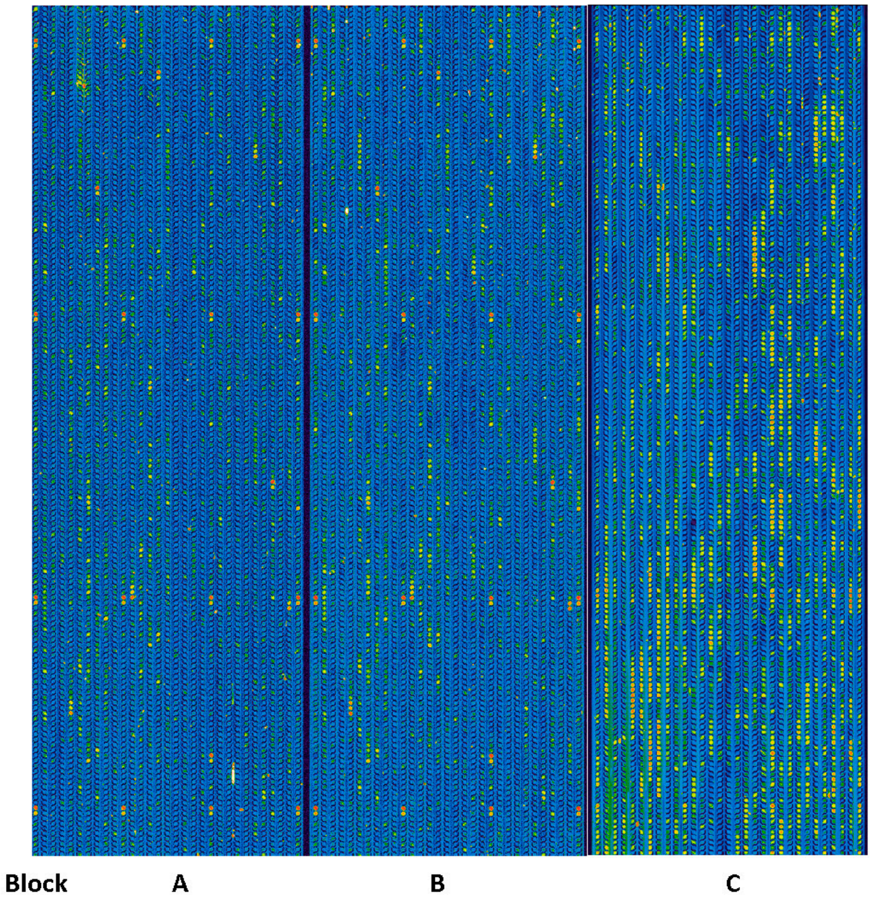

Figure S5. Raw images of peptide array. After binding assay (Block A-C for CsoS1, CsoS1D and RuBisCO, respectively), scanning of the chip was performed using microarray scanner PMT700 at 635 nm for signal of Alexa 647. Artificial color was applied to facilitate imaging of signal intensities with blue for no binding to red for maximal binding.

References

1. Scott, K.M.; Henn-Sax, M.; Harmer, T.L.; Longo, D.L.; Frame, C.H.; Cavanaugh, C.M. Kinetic isotope effect and biochemical characterization of form IA RubisCO from the marine cyanobacterium *Prochlorococcus marinus* MIT9313. *Limnol. Oceanogr.* **2007**, *52*, 2199–2204.
2. Dou, Z.; Heinhorst, S.; Williams, E.B.; Murin, C.D.; Shively, J.M.; Cannon, G.C. CO_2 Fixation kinetics of *Halothiobacillus neapolitanus* mutant carboxysomes lacking carbonic anhydrase suggest the shell acts as a diffusional barrier for CO_2. *J. Bio. Chem* **2008**, *283*, 10377–10384.
3. Cai, F.; Sutter, M.; Bernstein, S.L.; Kinney, J.N.; Kerfeld, C.A. Engineering bacterial microcompartment shells: Chimeric shell proteins and chimeric carboxysome shells. *ACS Synth. Biol.* **2014**, doi:10.1021/sb500226j.
4. Chen, X.S.; Casini, G.; Harrison, S.C.; Garcea, R.L. Papillomavirus capsid protein expression in *Escherichia coli*: Purification and assembly of HPV11 and HPV16 L1. *J. Mol. Biol.* **2001**, *307*, 173–182.
5. Baker, S.H.; Jin, S.; Aldrich, H.C.; Howard, G.T.; Shively, J.M. Insertion mutation of the form I *cbbL* gene encoding ribulose bisphosphate carboxylase/oxygenase (RuBisCO) in *Thiobacillus neapolitanus* results in expression of form II RuBisCO, loss of carboxysomes, and an increased CO2 requirement for growth. *J. Bacteriol.* **1998**, *180*, 4133–4139.
6. Kinney, J.N.; Salmeen, A.; Cai, F.; Kerfeld, C.A. Elucidating essential role of conserved carboxysomal protein CcmN reveals common feature of bacterial microcompartment assembly. *J. Biol. Chem.* **2012**, *287*, 17729–17736.
7. Heinhorst, S.; Cannon, G.C.; Shively, J.M. Carboxysomes and carboxysome-like inclusions. *Microbiol. Monogr.* **2006**, *2*, 141–165.
